# A Systematic Review of Food-Derived DNA Methyltransferase Modulators: Mechanistic Insights and Perspectives for Healthy Aging

**DOI:** 10.1016/j.advnut.2025.100521

**Published:** 2025-09-18

**Authors:** Manuela Campisi, Luana Cannella, Francesco Visioli, Sofia Pavanello

**Affiliations:** 1 Department of Cardiac, Thoracic, and Vascular Sciences and Public Health, University of Padua, Padua, Italy; 2 Department of Molecular Medicine, University of Padua, Padua, Italy; 3 Occupational Medicine Unit, University Hospital of Padua, Padua, Italy; 4 Centre of Studies and Activities for Space CISAS “G. Colombo” of University of Padua, Padua, Italy

**Keywords:** DNA methylation, DNMT inhibitors, dietary molecules, nutri-epigenetics, aging, biological age, natural extracts, anti-aging benefits, cancer prevention, personalized interventions

## Abstract

DNA methylation represents a crucial epigenetic mechanism orchestrating gene expression, cellular homeostasis, and the aging trajectory. Dysregulation of DNA methyltransferases (DNMTs)—the enzymes catalyzing this process—has been implicated in a wide spectrum of chronic conditions, including cancer, cardiovascular and metabolic disorders, and neurodegenerative diseases. Emerging evidence suggests that food-derived bioactive compounds can act as DNMT inhibitors, reshaping epigenetic landscapes. This systematic review, registered in PROSPERO (CRD42022320316), critically evaluated in vitro, in vivo animal, and ex vivo studies investigating the effects of dietary bioactives on DNMT expression and activity. A thorough search of PubMed up to 23 May, 2025, yielded 103 studies, of which 76 met the inclusion criteria. Eligible publications were original, peer-reviewed, and provided evidence from in vitro, in vivo animal, or ex vivo models. Frequently studied bioactives included epigallocatechin-3-gallate, curcumin, genistein, resveratrol, sulforaphane, and folate. Notably, nearly 90% of studies reported DNMT inhibition—often dose- and time-dependent. Approximately 21% defined minimal effective concentrations, predominantly for isolated compounds. Several studies described synergistic interactions between bioactives, and emerging data highlighted the gut microbiota’s mediating role in epigenetic modulation. Despite promising outcomes, the predominance of preclinical evidence and variability in experimental protocols and dosing limit the immediate translational impact. Nonetheless, current findings underscore the promise of dietary DNMT modulators as foundational elements for precision nutrition strategies aimed at promoting healthy aging and mitigating age-associated disease risk. The potential application of DNA methylation age as a biomarker of biological aging has been increasingly supported by recent literature, reinforcing its relevance in future nutritional epigenetics research. Further well-designed clinical trials are warranted to assess long-term efficacy, safety, and bioavailability of these compounds and to validate their use in personalized epigenetic interventions using biological aging markers. This review was funded by the European Union—Next Generation EU, PNRR Project Age-It (DM 1557 11.10.2022), and the University of Padua SID Grant (2024DCTV1SIDPROGETTI-00194).

## Graphical abstract

**Figure:**
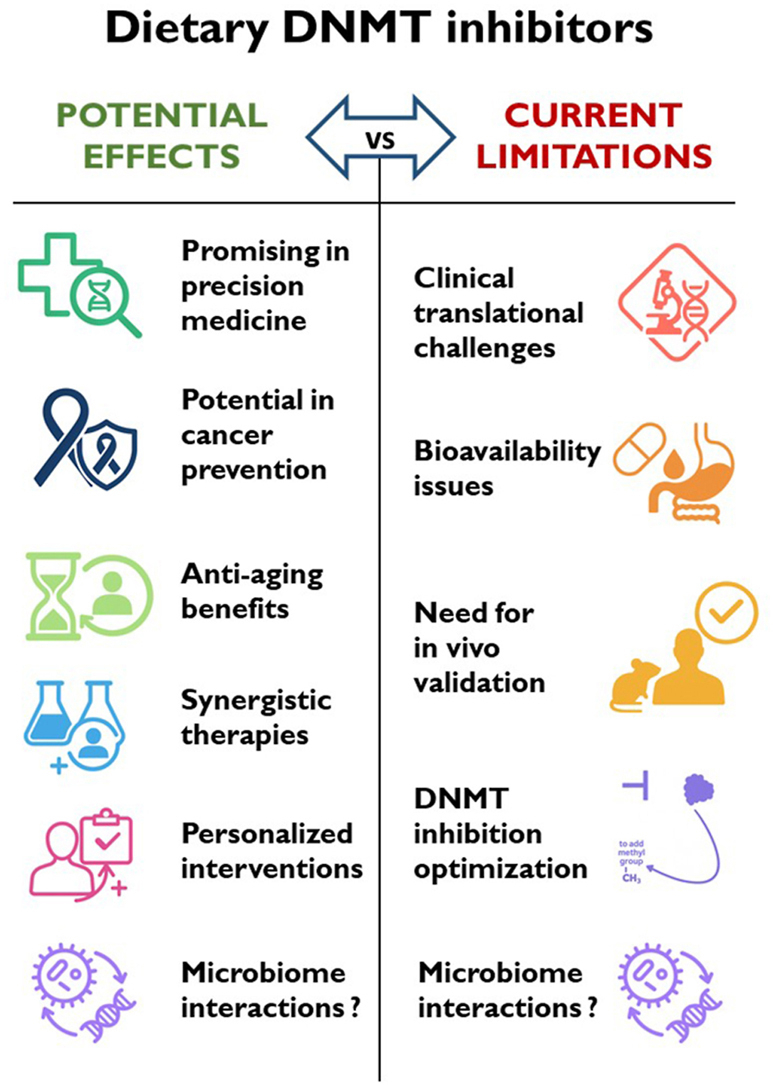

**Statement of Significance**

This systematic review is the first to comprehensively classify and evaluate food-derived DNMT modulators, highlighting their effects, synergistic combinations, and minimum effective doses. It bridges preclinical evidence with translational perspectives, paving the way for nutrition strategies targeting epigenetic aging and disease prevention.

## Introduction

Aging is a multifactorial biological process shaped by the interplay between genetic, environmental, and lifestyle-related factors [1]. Among these, nutrition has emerged as a modifiable determinant of healthspan and disease risk [1]. In the past decade, the field of nutritional epigenetics has gained momentum, offering mechanistic insights into how dietary components influence gene expression and cellular function without altering the DNA sequence. DNA methylation, one of the most extensively studied epigenetic modifications, involves the addition of a methyl group to cytosine residues within CpG sites. This reaction is catalyzed by DNA methyltransferases (DNMTs) [2]. This modification is essential for genome stability, gene silencing, and cellular differentiation, and its dysregulation and aberrant DNA methylation patterns have been implicated in cancer, neurodegeneration, cardiovascular and metabolic diseases, and age-related disorders [[2], [3], [4], [5], [6]].

There are 2 main classes of DNMTs: maintenance methyltransferases, primarily DNMT1, which ensures faithful inheritance of methylation patterns during DNA replication [7], and de novo methyltransferases, DNMT3A and DNMT3B, which establish new methylation patterns during early development [8]. Although the de novo enzymes are mostly active during embryogenesis, emerging proof suggests that they maintain residual activity in adult somatic tissues, contributing to ongoing epigenetic remodeling across the lifespan [8]. Modulation of DNMT activity is thus of considerable interest for identifying nutritional strategies aimed at slowing epigenetic aging and promoting healthy aging trajectories.

Recent evidence suggests that dietary bioactive compounds can influence DNA methylation by acting as natural DNMT inhibitors, modulating epigenetic markers and thereby affecting healthspan and disease susceptibility [[9], [10], [11]]. Polyphenols, flavonoids, isothiocyanates, and other plant-derived metabolites have demonstrated direct and indirect effects on DNMT activity, leading to global or locus-specific changes in DNA methylation patterns. For example, epigallocatechin gallate (EGCG) from green tea, genistein (GE) from soy, curcumin (CU) from turmeric, and resveratrol from grapes have been shown to reduce DNMT-mediated hypermethylation, thereby reactivating tumor suppressor genes and modulating inflammatory pathways [12]. Moreover, nutrients such as folate, choline, betaine, and vitamins B2, B6, and B12 regulate DNMT activity through modulation of S-adenosyl-L-methionine, the universal methyl donor in DNA methylation reactions [13]. Global hypomethylation and site-specific hypermethylation contribute to genomic instability, inflammation, and cellular senescence, suggesting that dietary DNMT modulators may have a crucial role in slowing epigenetic aging [7].

Emerging research also underscores the interconnection between diet, the gut microbiome, and epigenetic mechanisms, highlighting how microbial metabolites affect methyl donor availability and DNMT regulation, thus shaping age-associated epigenomic landscapes [14]. Beyond cancer [[15], [16], [17]], alterations in DNA methylation are now recognized as hallmarks of aging. These modifications in specific age-associated CpG sites are used in the calculation of DNA methylation age (DNAmAge), a reliable predictor of biological aging [18] and biomarker of age-related diseases [[19], [20], [21]]. In our previous works, we demonstrated that youthful epigenetic profiles can be reacquired through lifestyle interventions and that DNAmAge can effectively measure the impact of such strategies, including relaxation training [22] and natural supplementation with *Monarda didyma* L. [23]. In particular, our recent randomized clinical trial highlighted the geroprotective potential of *M. didyma* L. extract supplementation in aging workers, supporting the feasibility of dietary interventions to mitigate epigenetic aging [23]. These findings are in line with evidence from the DO-HEALTH study, in which a combined supplementation and physical activity protocol significantly improved markers of aging in older adults [24].

This review aims to systematically analyze recent findings on the epigenetic effects of dietary bioactives on DNMT regulation, with a particular focus on their mechanisms of action, efficacy, and clinical relevance. Specifically, we will:

1) Identify food-derived bioactive compounds —including bioactive dietary molecules, natural extracts, and their combinations—with demonstrated capacity to modulate DNA methylation by direct or indirect DNMT inhibition or regulation.
2) Evaluate the minimum effective concentrations (MECs) of these food-derived compounds required to induce DNMT inhibition and epigenetic changes in preclinical and clinical models.

By bridging emerging evidence in nutritional epigenetics with translational aging research, this review aims to elucidate the potential of food-derived and dietary DNMT modulators as a basis for future personalized nutrition strategies to promote healthy aging and prevent age-related diseases.

## Methods

### Search strategy

**FIGURE 1:**
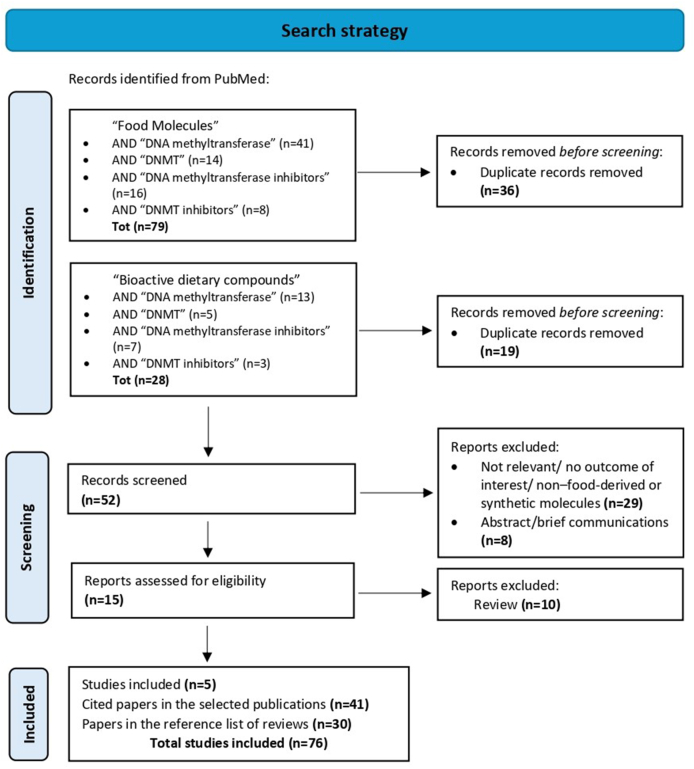
PRISMA flow diagram of the systematic literature search, including search strategy and screening process. DNMT, DNA methyltransferase.

This systematic review was conducted according to the PRISMA guidelines [25]. PubMed database was used as the search engine to identify studies investigating the molecular mechanisms by which dietary compounds and food-derived molecules influence DNA methylation patterns and regulate gene expression by direct epigenetic effects on DNA methylation, specifically through the inhibition of DNMT enzymes. Keywords used in the search strategy were “food molecules” or “bioactive dietary compounds,” each combined with “DNA methyltransferase,” “DNMT,” “DNA methyltransferase inhibitors,” or “DNMT inhibitors,” as reported in Supplemental Table 1. This search strategy displayed relevant articles published up to 23 May, 2025. This study was registered in PROSPERO as CRD42022320316. Figure 1 shows the PRISMA flow diagram including search strategy with the key terms and the screening process according to the PRISMA guidelines [25]. We found *n* = 107 studies related to the key terms used, and after the screening process, also considering the additional articles cited in the selected publications (*n* = 41) and in the reference lists of relevant reviews (*n* = 30), we identified a total of *n* = 76 studies that met the inclusion criteria, reported in Table 1. All the studies included were carried out between 2003 and 2024.

**TABLE 1:** Nutrients and molecules with epigenetic effects on DNMT.

Reference	Dietary molecule tested	Experimental models adopted	Dose of food molecule tested	Methods	Mechanism of action	Main results	Main source
**In vitro studies**							
[26]	BBR	In vitro: HT29 colorectal adenocarcinoma cells	Treatment with 280 μmol/L of BBR for 48 h	qRT-PCR to analyze DNMT1, DNMT3A, and DNMT3B mRNA levels	BBR can change the intestinal microbiota, potentially regulating the expression of DNMTs.	↓ mRNA expression levels of DNMT1 and DNMT3B ↔ DNMT3A	Chinese herbal medicine-derived alkaloid
[27]	Black seed oil Nigella sativa oil, with its pharmacologically active compound, TQ	In vitro*:* Jurkat, MCF-7, and HeLa cells	0.6 and 1.25 of black seed oil (v/v) and different concentrations of TQ from a stock solution of 10 mM for 24 h	qRT-PCR to analyze DNMT expression	TQ may interact with DNMT1 indirectly through its interaction with the SRA domain of UHRF1 during DNA replication and directly with the catalytic pocket of DNMT1 by inhibiting the activity of DNMT1 and inducing global DNA hypomethylation.	↓ mRNA expression of DNMT1 in a dose-dependent manner in all cell lines treated with either black seed oil or with pure TQ compared with the control, with consequent inhibition of cell proliferation and induction of apoptosis in cancer cells	Black seeds
[28]	SFN, GE, and NaB	In vitro: MDA-MB-231 and MCF-7 breast cancer cell lines	Treatment with single, double, and triple combinations of 5 μM SFN, 15 μM GE, and 2.5 mM NaB: SFN + GE; SFN + NaB; GE + NaB; SFN + GE + NaB	qRT-PCR to analyze *DNMT3A* and *DNMT3B* gene expression. Western blot to analyze DNMT3A and DNMT3B protein levels. DNMT activity assay kit (Epigentek)	—	↓ *DNMT3A* and *DNMT3B* gene expression and protein level with combination of GE and NaB, and of all dietary compounds (SFN-GE-NaB) in MDA-MB-231 cells ↓ DNMT3A levels with combination of SFN and GE, GE and NaB, and of all compounds (SFN-GE-NaB) in MCF-7 cells ↓ DNMT activity with combination of SFN and NaB, and of all dietary compounds (SFN-GE-NaB) in MDA-MB-231 cells ↓ DNMT activity with combination of SFN and GE, GE and NaB, and all compounds in MCF-7 cells	SFN: cruciferous vegetables NaB (short-chain fatty acid produced by gut microbiota): dietary supplementation GE: soy
[29]	Hop extract and its bioactive compound 6-PN	In vitro: MCF-7 cells	Treatment with 5 μg/mL hop extract or 1 μM of 6-PN for 12 h	ChIP assay and qPCR	Methylation and downregulation of *CYP1A1* by DNMT1 and ERα is reversed through AhR activating 6-PN and hop extract.	↓ DNMT1-associated *CYP1A1* with hop extract or 6-PN treatment ↔ Effect not observed using anti-DNMT3B antibody	*Humulus lupulus* L., Cannabaceae
[30]	Methanolic extract of *Paederia foetida* leaves and its phytochemicals including alkaloids, flavonoids, terpenoids, steroids, cardiac glycosides, as well as the presence of lupeol and β-sitosterol.	In vitro: Human prostate cancer cell lines PC-3, DU-145, and normal human keratinocytes, HaCaT	Different concentrations of methanolic extract of *Paederia foetida* leaves for 24 and 72 h	DNMT activity Assay kit (Epigentek) Western blot to analyze protein levels	—	↓ DNMT activity in cells treated with methanolic extract of *Paederia foetida* leaves in a dose-dependent manner ↓ Protein level expression of DNMTs in treated cells when compared with untreated one	*Paederia foetida* or Ghandhali (family of Rubiaceae, from India)
[31]	GE	In vitro: Human cervical carcinoma HeLa cells	Treatment with 50 μM GE for 48 h	DNMT activity assay kit (Epigentek) qRT-PCR for DNMT expression	—	↓ DNMT1, DNMT3B, and DNMT3A expression ↓ DNMT activity by 48% in comparison with the untreated control	Soybeans
[32]	Quercetin	In vitro: Human cervical carcinoma HeLa cells	Treatment with 25 and 50 μM quercetin for 48 h	DNMT activity Assay Kit (Epigentek) qRT-PCR to analyze DNMT expression	Quercetin may competitively inhibit DNMT3A and DNMT3B. binding the polycomb repressor protein EZH2 may inhibit its ability to recruit DNMT. Quercetin promotes a decrease in PI3K and WNT activity. PI3K-AKT and WNT pathways stabilize DNMT1, contributing to DNA methylation	↓ DNMTs activity in a dose-dependent manner (25 and 50 μM) by 32% and 49%, respectively, compared with untreated control ↓ DNMTs expression with quercetin 25 and 50 μM	Onions, grapes, berries, cherries, broccoli, and citrus fruits
[33]	GA	In vitro: Human non-small cell cancer cell lines A549 and H1299	Treatment with 10 μM GA for 7 d	Western blot to analyze DNMT1 protein levels	GA exhibits an inhibitory effect on DNMT1 activity possibly by negatively regulating Akt phosphorylation, in turn reducing both the nuclear import and protein stability of DNMT1.	↓ Nuclear DNMT1 and DNMT3B almost disappeared after GA treatment (10 μM) for 7 d ↓ Cytoplasmic DNMT1 also observed in GA-treated (10 μM) of H1299 cells for 7 d ↓ In both the nuclear and cytoplasmic abundance of DNMT1 after either short-term (120 h) or long-term (240 h) treatment with GA (10 μM)	Gallnuts, sumac, witch hazel, tea leaves, oak bark
[34]	Vitamin E	In vitro: human colorectal adenocarcinoma cell line Caco-2	Treatment with 10 or 50 μM vitamin E. Composition = d-α-tocopherol 20 IU/mL, other tocopherols 15 mg/ml, and tocotrienols 2 mg/ml.	qRT-PCR to analyze DNMT1 expression	—	↑ DNMT1 expression after treatment with 10 μM vitamin E ↔ 50 μM showed no effect ↑ At hyperglycemic conditions, both vitamin E concentrations increased DNMT1 expression, which was significant at 10 μM	—
[35]	SFN	In vitro: Human breast cancer MCF-7, MDA-MB-231, and SK-BR-3 cells	Treatment with 5, 10, and 20 μM of SFN for 24 h	qRT-PCR to analyze DNMT expression. Western blot to analyze DNMT1, DNMT3A, and DNMT3B protein levels	—	↓ DNMT3A mRNA and protein levels in MDA-MB-231 cells treated with SFN 10 μM ↓ DNMT1 and DNMT3B protein levels ↔ mRNA levels of DNMT1 and DNMT3B	—
[36]	DS	In vitro*:* Human breast cancer MCF-7 and MDA-MB-231 cells	Treatment with 1.15–5.76 μM DS for 72 h	qRT-PCR to analyze DNMT1, DNMT3A, and DNMT3B mRNA	—	↔ DNMT1, DNMT3A, and DNMT3B mRNA in MCF-7 cells after 1.15–5.76 μM DS treatment ↓ DNMT3A mRNA in MDA-MB-231 cells with DS at 5.76 μM DS	Wld yam (*Dioscorea villosa*), *Saponaria officinalis*, *Quillaja saponaria*, *Sapindus mukorossi*
[37]	PEITC	In vitro*:* Human prostate cancer LNCaP cells	Treatment with PEITC (1–10 μM) for 1 and 5 d	qRT-PCR to analyze DNMT1, DNMT3A, and DNMT3B expression Western blot to analyze DNMT3A and DNMT3B protein levels	—	↓ DNMT1, DNMT3A, and DNMT3B mRNA in cells treated with PEITC (5 μM) ↓ Protein levels of all DNMTs in cells treated with 2.5 μM or 5 μM PEITC compared with control cells	Cruciferous vegetables such as broccoli and watercress
[38]	Resveratrol and pterostilbene	In vitro: Breast cancer MDA-MB-157 cell lines	Treatment with 15 μM resveratrol and 5 μM pterostilbene alone as well as in combination, for 72 h	DNMT activity Assay kit (Epigentek)	—	↓ DNMT activity in MDA-MB-157 breast cancer cells after combination treatment ↓ DNMT activity after 5 μM of pterostilbene, but less than the combination treatment	Resveratrol: grapevines, red grapes, mulberries, peanuts, and pines Pterostilbene: blueberries and grapes
[39]	Emodin	In vitro: Human pancreatic cancer PANC-1 cells	Treatment with 40 μM and in combination with 5-Aza-dC (1 μM), for 72h.	FQ-PCR to analyze DNMT1, DNMT3A, and DNMT3B expression Western blot to analyze DNMT1, DNMT3A, DNMT3B protein levels	—	↓ mRNA expression levels and protein levels of DNMT1 and DNMT3A in cells treated with emodin and in combination with 5-Aza-dC ↔ mRNA expression levels of DNMT3B	Rhubarb
[40]	PepE	In vitro: Lung cancer A549 cells	Treatment with PepE at various concentrations (0–100 μM) for 48 h PepE IC_50_ value was 2.177 μM	DNMT activity Assay kit (Epigentek) qRT-PCR to analyze DNMT1 expression Western blot to analyze DNMT1 protein levels	PepE directly interacts with the active domain of DNMT1, potentially affecting its activity. This result indicated that the transcriptional repression activity might be owing to its ability to reduce the expression of NF-κB (p65) and Sp1 and the reduction in the binding of transcription factors to the *DNMT1* promoter.	↓ DNMT activity of A549 cells in a dose-dependent manner, with IC_50_ value 0.205 μM Dose-dependent depletion of DNMT1 protein levels and ↓ mRNA levels in a dose-dependent manner, at 4.0 and 8.0 μM doses	*Peperomia dindygulensis*
[36]	WYRE	In vitro: Human breast adenocarcinoma MCF-7 and MDA-MB-231 cells	Treatment with 10, 25, and 30 μg/mL of WYRE for 72 h	qRT-PCR to analyze DNMT1, DNMT3A, and DNMT3B expression	—	↑ DNMT1, DNMT3A, and DNMT3B mRNA in MDA-MB-231 cells treated with 10, 25, and 30 μg/mL WYRE ↔ DNMT1, DNMT3A, and DNMT3B mRNA in MCF-7 cells	Wild yam root
[41]	Resveratrol and pterostilbene	In vitro: Breast cancer CC1806 and MDA-MB-157 cells and control MCF10A breast epithelial cells	Treatment with resveratrol (15 μM), pterostilbene (5 μM), and their combination for 72 h	DNMT activity Assay Kit (Epigentek) qRT-PCR to analyze DNMT expression	—	↓ DNMT1, DNMT3A, and DNMT3B at the transcription level, as well as enzymatic activity in HCC1806 cells after 72 h of combination treatment ↔ DNMT enzyme activity in MCF10A control cells after 72 h of combination treatment ↓ DNMT activity and gene expression after 72 h of treatment with resveratrol (15 μM) or pterostilbene (5 μM)	Resveratrol: grapevines, red grapes, mulberries, peanuts, and pines Pterostilbene: blueberries and grapes
[42]	EGCG	In vitro: Human cervical carcinoma HeLa cells	HeLa cells were treated with EGCG (25 μM) and 5-Aza-dC as positive control (1 μM) for 3 d	DNMT activity Assay kit (Epigentek) qRT-PCR to analyze DNMT3B expression	EGCG directly binds in the substrate binding pocket of DNMT3B.	↓ Enzymatic activity of DNMT in cells treated in time-dependent manner ↓ Expression of DNMT3B in a time-dependent manner in cells treated with 25 μM EGCG (24, 48, and 72 h)	Green tea, white tea, black tea
[43]	SFN	In vitro: Human cervical carcinoma HeLa cells	Treatment with 2.5 μM SFN for 0, 24, 48, and 72 h	DNMT activity assay kit (Epigentek) qRT-PCR to analyze DNMT3B expression	SFN directly binds in the substrate binding pocket of the enzymes. A very high proportion of SFN was observed in the defined substrate binding pocket of mDNMT3B, preventing the entry of the natural ligand into the active site.	↓ DNMT activity (7%, 15%, and 23%) in cells compared with the untreated control in a time-dependent manner ↓ DNMT3B expression in a time dependent-manner (24, 48, and 72 h) in HeLa cells compared with the untreated control	Cruciferous vegetables such as broccoli, cabbage, garden cress, cauliflower, and Brussels sprouts
[44]	Quercetin and isoliquiritigenin	In vitro*:* Gastric carcinoma SNU-719 cell line	Treatment with 62 μM isoliquiritigenin and 45 μM quercetin	qRT-PCR to analyze DNMT expression Western blot to analyze DNMT protein levels	DNMT1 is regulated by quercetin in a STAT3-independent manner, and DNMT3A is controlled by quercetin in a STAT3-dependent manner.	↓ Quercetin treatment almost completely eliminated both DNMT1 and DNMT3A expression ↔ DNMT1 and DNMT3A protein expression with isoliquiritigenin treatment	Quercetin: vegetables, fruits, nuts, tea, red wine, and propolis Isoliquiritigenin: licorice
[45]	SFN	In vitro*:* Human breast cancer MCF-7 and MDA-MB-231 cells	Treatment with 10 and 22 μM	qRT-PCR to analyze DNMT1 expression	—	↔ DNMT1 expression in MCF-7 treated cells	Cruciferous vegetables
[46]	EGCG	In vitro: Human colon cancer HT-29 and HCT-116 cell lines	Treatment with 50, 100, 150 μM EGCG for 48 and 72 h	qRT-PCR to analyze DNMT3A, DNM3B, and DNMT1 expression Western blot to analyze DNMT3A protein levels Western blot to quantify DNMT3A protein degradation	Direct binding between EGCG and DNMTs EGCG reduces the association between DNMT3A and the E3 ubiquitin ligase, UHRF1, in methylation-sensitive HCT-116 cells.	↓ mRNA levels of DNMT3A in both HCT 116 and HT-29 cell lines ↓ DNMT3B transcript in HCT-116 cells at 150 μM EGCG ↓ All studied DNMT transcripts in HT-29 cells at 48 h at all EGCG concentrations ↓ All studied transcripts at 150 μM ECGG in HCT-116 at 72 h, but more drastic ↓ in expression of all DNMT in HT-29 cells ↓ DNMT3A protein levels in the HCT-116 cell line in a time-dependent manner ↔ DNMT3A protein levels at 48 h in the HT-29 cells, with a noticeable ↓ in expression at 150 μM at 72 h	Green tea
[47]	EGCG and procyanidin B2	In vitro: Human breast cancer MDA-MB-231 cell line	Treatment with EGCG and procyanidin B2 at increasing concentrations (1, 2.5, 5, 10, and 15 μM)	DNMT activity Assay kit (Epigentek)	Procyanidin B2–3, 3′-di-*O*-gallate moiety presents itself as a valid inhibitor having strong correlation with the active site residues of DNMTs, as well as the EGCG.	Procyanidin B2 attenuates DNMT1 and DNMT3A activity) at IC_50_ 6.88 ± 0.647 μM EGCG attenuates DNMT activity at IC_50_ 9.36 ± 1.02 μM	EGCG: Tea Procyanidin B2: in proanthocyanidin extracts from grape seed
[48]	β-elemene	In vitro: NSCLC (A549 and PC9) lung cancer cells	Treatment with 0, 5, 10, 20, 40, 60 μg/mL for 24 h	Western blot to analyze DNMT1 protein levels	β-elemene inhibits NSCLC cell growth via ERK1/2- and AMPKα-mediated inhibition of transcription factor Sp1, followed by reduction in DNMT1 protein expression.	↓ Protein expression of DNMT1 in a dose-dependent manner in both A549 and PC9 and inhibited cell growth	*Rhizoma zedoariae*
[49]	GA	In vitro*:* Human cardiovascular endothelial cell (EAhy926) and human cerebrovascular endothelial cells (HBEC-5i)	EAhy926 endothelial cells were pretreated with GA (10 and 100 μM) for 4 h and then exposed to DL-Hcy, Ado, and TNF for 20 h	qRT-PCR to analyze DNMT1 expression Western blot to analyze DNMT1 protein levels	GA may inhibit protein degradation to attenuate DNMT1 protein depletion.	GA pretreatment restored DNMT1 expressions after treatment with DL-Hcy-Ado-TNF as opposed to the ↑ expression of DNMT1 protein	Tea, grapes, and pomegranate
[50]	API	In vitro: JB6 P+ epidermal keratinocytes	Treatment with 1.56 and 6.5 μM API for 5 d	Western blot to analyze DNMT1, DNMT3A, and DNMT3B protein levels	—	↓ Expression of all DNMT proteins, especially DNMT1 and DNMT3B, at the higher dose	*Matricaria recutita* L.
[51]	EGCG and NaB	In vitro: colorectal cancer RKO, HCT-116, and HT-29 cells	Treatment with 10 μM EGCG and NaB 5 mM for 48 h	Western blot to analyze DNMT1, DNMT3A, and DNMT3B protein expression qRT-PCR to analyze DNMT1 expression	NaB decreased DNMT1 levels via proteasomal degradation. Combination of EGCG and NaB significantly reduced DNMT1 levels; may be due to the combination of inhibiting the catalytic site along with proteasomal degradation.	↓ DNMT1 expression and protein levels in all 3 cell lines for the combination treatment compared with EGCG and NaB alone ↓ DNMT3A and DNMT3B levels in the combination treatment compared with the controls	EGCG: green tea NaB: dietary microbial fermentation product of fiber
[52]	SFN	In vitro: JB6 P+ (JB6 Cl 41-5a, ATCC CRL-2010) skin fibroblast cells	Treatment with 1.0–5.0 μmol/L for 5 d	Western blot to analyze DNMT1, DNMT3A, and DNMT3B protein levels	—	↓ DNMT1, DNMT3A, and DNMT3B protein expression after SFN treatment (1.0–5.0 μmol/L) in a concentration-dependent manner in JB6 P+ cells after 5 d of treatment	Cruciferous vegetables, including broccoli, cabbage, cauliflower, Chinese cabbage, and watercress
[53]	GE	In vitro: Human breast cancer MCF-7 and MDA-MB-231 cells	Treatment with 60 μM or 100 μM GE for 24, 48, and 72 h	qRT-PCR to analyze DNMT1 expression Western blot to analyze DNMT1, DNMT3A, and DNMT3B protein levels DNMT Activity Assay Kit (Epigentek)	GE might directly interact with the catalytic domain of DNMT1, thus competitively inhibiting the binding of hemimethylated DNA to the catalytic domain of DNMT1.	↓ DNMT1 expression and protein levels in both MCF-7 and MDA-MB-231 cells in a time- and dose-dependent manner ↔DNMT3A and DNMT3B mRNA expression and protein levels ↓ DNMT activity in both MCF-7 and MDA-MB-231 cells in a time- and dose-dependent manner	Soybean and fava bean
[54]	Mahanine	In vitro: Human prostate cancer PC3 and LNCaP cells	Treatment with mahanine 10μM for 24 h Treatment with mahanine 10 μM and 20 μM for 24–48 h	Immunofluorescence staining for the cellular localization of the 3 DNMTs Western blot to analyze DNMT1, DNMT3A, and DNMT3B protein levels qRT-PCR to analyze DNMT1, DNMT3A, and DNMT3B expression	Mahanine treatment induces the degradation of DNMT1 and DNMT3B but not DNMT3A via the ubiquitin-proteasome pathway with a decline in phospho-Akt levels and a disruption in the interaction of Akt with DNMT1 and DNMT3B.	↓ Nuclear levels of DNMT1 and DNMT3B after treatment with 10 μM mahanine for 24 h, with a noticeable ↓ in its cytoplasmic staining as well. ↔ Cellular distribution of DNMT3A ↓ DNMT1 and DNMT3B protein levels in a dose-dependent manner in both cell lines treated with 10 μM and 20 μM for 24 to 48 h ↔ DNMT3A protein levels ↔ DNMT1 and DNMT3B mRNA levels in both cell lines at 20 μM and 10 μM, respectively, for 24 h, suggesting that the decline in DNMT1 and DNMT3B protein levels occurs posttranslationally	*Micromelum minutum*
[55]	EGCG and SFN	In vitro: ovarian cancer SKOV3 cells	Treatment with EGCG 20 μmol/L, SFN 10 μmol/L, and their combination	Western blot to analyze DNMT1 protein levels	—	↓ DNMT1 level after combination treatment with EGCG and SFN	EGCG: green tea SFN: cruciferous vegetables
[56]	EGCG, GE, CU, resveratrol, withaferin A, and guggulsterone	In vitro: Human breast carcinoma MCF7 and MDA-MB-231 cell lines.	Treatment with EGCG, GE, withaferin A, CU, resveratrol, guggulsterone (equivalent to the IC_50_ of each compound), and decitabine (at 10 μM for MDA-MB-231 cells and 12 μM for MCF7 cells) for 96 h	qRT-PCR to analyze DNMT1, DNMT3A, and DNMT3B expression Western blot to analyze DNMT1 protein level	CU covalently blocks the catalytic thiolate of DNMT1 to exert its inhibitory effect.	↓ Transcript levels of all DNMTs in both cell lines after treatment 2- to 3-fold ↓ in DNMT1 protein levels observed in MCF7 and MDA-MB 231 cells	Turmeric
[57]	RAS, including its major constituent ligand	In vitro: Prostate cancer TRAMP C1 cells	Treatment with Lig (50 μM) or RAS (8.5 μg/mL) for 3 d	qRT-PCR to analyze DNMT expression. Western blotting to analyze DNMT protein levels	—	↔ Lig (0–50 μM) and RAS (0–8.5 μg/mL) did not affect either the mRNA or protein expression levels of DNMT1, DNMT3A, and DNMT3B compared with control cells	RAS, also known as “Danggui,” an edible and medicinal herb from Asia
[58]	SFN	In vitro: Prostate cancer TRAMP C1 cells	Treatment with 1.0, 2.5, and 40 μM SFN for 5 d	Western blot to analyze protein levels	SFN may interfere with the formation of a transcriptional regulator complex, consisting of methyl-CpG binding proteins, DNMT, and HDAC.	↓ Protein levels of DNMT1 and DNMT3A in a dose-dependent manner ↔ No significant alteration of DNMT3B expression	Cruciferous vegetables
[59]	SFN	In vitro: porcine satellite cells	Treatment with 5, 10, and 15 μM for 48 h	qRT-PCR to analyze DNMT1 expression	—	↓ DNMT1 after treatment with 10 μM and 15 μM of SFN	Cruciferous vegetables
[60]	TCS	In vitro*:* Human cervical adenocarcinoma (HeLa) and human cervical squamous carcinoma (CaSki) cells	Treatment with various concentrations (0, 20, 40, and 80 μg/mL) TCS for 48 h	qRT-PCR to analyze DNMT1 expression Western blot to analyze DNMT1 protein level DNMT1 enzyme activity assay kit (Epigentek)	TCS is a type I ribosome inactivating protein (RIP); we suggest that TCS downregulation of the DNMT expression and enzyme activity may correlate with this mechanism of ribosome inactivating protein.	↓ DNMT1 mRNA expression after treatment with various concentrations (0, 20, 40, and 80 μg/mL) of TCS for 48 h in both cell lines ↓ Protein expression after treatment with TCS in a dose-dependent manner ↓ DNMT1 enzyme activity (31.3% and 56.7%) in cells treated with 20 and 40 μg/ml	Root tubers of the Chinese medical herb *Trichosanthes kirilowi*
[61]	GTPs including EC, ECG, EGC, and EGCG, and SFN	In vitro: Human breast cancer MCF-7, MDA-MB-453, and MDA-MB-231 cells	Treatment with 10 μM SFN 20 μM GTPs, 20μM GTPs+ 5 μM SFN 20 μM GTPs+ 10 μM SFN, for 3 d	Western blot analysis for DNMT1, DNMT3A, DNMT3B protein levels DNMT activity Assay kit (Epigentek) ELISA	Direct inhibition of DNMT by EGCG GTPs could lead to a decrease in available SAM and an increase in SAH and homocysteine levels, thereby providing evidence of an indirect inhibition of DNA methylation by EGCG/GTPs. Combinations of GTPs and SFN induced the disruption of the transcriptional repressor multimolecular complex, HDAC1/DNMT1/SUV39H1.	↓ DNMT activity, expression, and DNMT3A protein levels after GTP treatment (20 μM) in MDA-MB-231 cells ↓ DNMTs activity and DNMT1 protein levels after SFN treatment (10 μM) in MDA-MB-231 cells Combinations of GTPs and SFN (20 +5 μM and 20 + 10 μM) have more pronounced DNMT-inhibitory effects and ↓ DNMT1, DNMT3A, and DNMT3B protein levels	Green tea
[62]	GSPs	In *vitro* and in *vivo* models: Skin cancer A431 and SCC13 cells	Treatment with various concentrations of GSPs (0, 5, 10, 15, and 20 μg/mL) for 5-Aza-dC for 3 or 5 d	DNMT activity Assay kit (Epigentek) qRT-PCR to analyze DNMT1, DNMT3A, DNMT3B expression Western blot to analyze DNMT1, DNMT3A, DNMT3B protein levels	—	↓ DNMT activity in A431 (15%–78%) and SCC13 (15%–80%) cells after treatment for 5 d compared with cells treated for 3 d, with a dose-dependent effect ↓ mRNA levels of DNMT1, DNMT3A, and DNMT3B in both A431 (50%–70%) and SCC13 (60%–70%) cells after treatment with GSPs for 5 d ↓ Protein expression levels of DNMT1, DNMT3A, and DNMT3B after treatment with GSPs of A431 and SCC13 cells for 5 d compared with non-GSP-treated controls	Grape seed
[63]	SFN	In vitro: BPH-1 cells, androgen-dependent prostate cancer epithelial cells (LNCaP), and androgen-independent prostate cancer epithelial cells (PC3)	Treatment with SFN (15 or 30 μM) for 24 or 48 h	qRT-PCR to analyze mRNA expression levels of DNMTs (DNMT1, DNMT3A, DNMT3B) Western blot to analyze DNMT1 protein levels	SFN may act as a dual epigenetic regulator by inhibiting DNMTs. In addition to transcriptional regulation of DNMTs, alternative posttranscriptional and posttranslational regulations of DNMTs could be targeted by SFN.	↓ DNMT1 and DNMT3A mRNA expression at both treatment doses in BPH-1 and PC3 cells ↔ DNMT1 protein expression in BPH-1 and PC3 cells ↓ DNMT1 and DNMT3B mRNA expression ↓ DNMT1 protein expression in LNCaP cells	Cruciferous vegetables: broccoli, BSp, and brussels sprouts
[64]	EGCG	In vitro: Breast cancer MCF-7 and MDA-MB-231 cells and normal control MCF10A cells	Treatment with 40 μM for 6 and 9 d	DNMT activity Assay kit (Epigentek)	EGCG inhibition of DNMT activity might be because of the direct binding of EGCG to the active site of DNMTs.	↓ DNMT activity after EGCG treatment at 6 and 9 d in breast cancer cells	Green tea
[65]	EGCG	In vitro: Human epidermoid carcinoma A431 and SCC13 cells	Treatment for 3 or 6 d with various concentrations (10, 15, and 20 μg/mL) of EGCG or other catechins, such as EC, EGC, and ECG	DNMT activity Assay Kit (Epigentek) qRT-PCR to analyze DNMT1, DNMT3A, DNMT3B expression Western blot to analyze DNMT1, DNMT3A, and DNMT3B protein levels	—	↓ DNMT activity greater after treatment for 6 d than for 3 d, in a concentration-dependent manner in both A431 and SCC 13 cells ↓ mRNA levels of DNMT1, DNMT3A, and DNMT3B in A431 cells after treatment for 3 d and even more for 6 d ↓ Protein levels of DNMT1, DNMT3A, and DNMT3B after treatment for 6 d compared with non-EGCG-treated controls	Green tea
[66]	CU	In vitro: Prostate cancer LNCaP cell lines	Treatment with 5 μM CU	Western blot to analyze protein levels	—	↔ Protein expression levels of DNMT1 and DNMT3A	Turmeric curry, *Curcuma longa*
[67]	EGCG	In vitro: Jurkat T cells	Treatment with 2, 10, and 50 μM EGCG	qRT-PCR to quantify DNMT1, DNMT3A, and DNMT3B expression	EGCG directly binds to the substrate binding pocket of DNMT.	↓ DNMT1, DNMT3A, and DNMT3B expression after treatment with 10 μM or 50 μM	Green tea
[16]	GE	In vitro: Human prostate carcinoma cell lines (LNCaP, PC3) and a normal epithelial prostate cell line (RWPE-1)	Treatment with 0, 10, 25, and 50 μmol/L GE	Enzymatic activity measured calorimetrically through an ELISA-like reaction Epiquik DNMT1, DNMT3A, and DNMT3B Assay kits (Epigentek)	—	↓ DNMT activity by treatment with 50 μM GE in LNCaP and PC3 cells ↓ Levels of DNMT1 proteins in GE-treated samples compared with untreated controls	Soybeans
[15]	SFN	In vitro: Breast cancer MCF-7 and MDA-MB-231 cells and normal control MCF10A cells	Treatment with 0, 5, 10, 15, and 20 μmol/L SFN for 15 d	Western blot to analyze DNMT1, DNMT3A, and DNMT3B protein levels Colorimetric assay kit (Epigentek) and ELISA to analyze DNMT activity	—	↓ DNMT1 and DNMT3A expression after treatment with 10 μM SFN in 6 d in MCF-7 cells by 62% and 81%, respectively, and in MDA-MB-231 cells, by 48% and 78%, respectively	Cruciferous vegetables: broccoli, BSp, and brussels sprouts
[68]	Baicalein, myricetin, protocatechuic acid, phloretin, sinapic acid, syringic acid, resveratrol, rosmarinic acid, ellagic acid, betanin, cyanidin, and galangin	In vitro*:* Breast cancer MCF7 cells	Treatment at 20 μM or 40 μM for 3 d	DNMT activity assay kit (Epigentek) Western blot to analyze DNMT1 protein levels	Noncompetitive inhibition of DNA methylation catalyzed by DNMT largely due to the increased formation of SAH resulting from the COMT-mediated *O*-methylation of catechol-containing polyphenols, particularly ellagic and rosmarinic acids.	↓ DNMT activity with all phytochemicals, with betanin being the weakest, whereas rosmarinic and ellagic acids were the most potent inhibitors (≤88% inhibition) ↔ DNMT1 transcript and protein levels ↓ DNMT1 protein levels after treatment with rosmarinic acid in MCF7 cells at both concentrations (20 and 40 μM) by ∼30% and 20%, respectively	—
[69]	GTPs, including EGCG	In vitro: Human prostate cancer LNCaP cells	Treatment with 1–20 μg/mL of GTP Polyphenon E (Mitsui Norin) and 5–20 μM EGCG for 3, 7, and 14 d	DNMT activity Assay Kit (Epigentek) qRT-PCR to analyze DNMT1 expression Western blot to analyze DNMT1, DNMT3A, and DNMT3B protein levels	EGCG has been shown to inhibit DNMT activity by blocking the entry of cytosine nucleotide into the active site by forming hydrogen bonds with 2 critical residues, Glu1265 and Pro1223, in the catalytic pocket of DNMT. GTP not only inhibits the catalytic activity of DNMT1 but also downregulates its protein level.	↓ DNMT activity (16%, 28%, and 56%) in a dose-dependent manner in LNCaP cells after 7 d exposure to 5, 10, and 20 μM EGCG. Higher inhibition in DNMT activity (36%, 62%, and 78%) after 5, 10, and 20 μg/mL GTP exposure, compared with untreated control. ↓ DNMT1 mRNA levels in a dose-dependent manner, by 0.5- to 0.85-fold, in LNCaP cells treated with varying doses of GTP (1–10 μg/mL) for 3 d, as well as when treated with 10 μg/mL GTP from 1–14 d, with 0.7- to 0.95-fold inhibition ↓ Nuclear fraction of DNMT protein in a dose-dependent fashion after treatment with GTP, with complete inhibition in 14 d ↔ DNMT3A and DNMT3B protein levels	Green tea
[70]	Resveratrol	In vitro: Human breast cancer MCF-7 cells	Pretreatment with TCDD 100 nmol/L or TCDD + resveratrol for 12 h was followed by cotreatment for 24 h with E2, E2 + TCDD, or E2 + TCDD + resveratrol (5, 10, and 20 μM)	Western blot to analyze DNMT1 protein levels	Resveratrol is a natural AhR antagonist. The cotreatment as well as pretreatment with resveratrol antagonized the recruitment of DNMT1.	↓ DNMT1 protein levels by cotreatment or pretreatment with resveratrol at the BRCA-1 promoter in cells treated with TCDD	Grapes, berries, peanuts, and pines
[71]	Folate	In vitro: Primary cultured tumor U373 cells	Folate (4 or 40 μg/mL) ± antioxidant cocktail (20 μmol/L β-carotene; 200 μmol/L ascorbic acid) for 7 d	qRT-PCR to analyze DNMT expression	Folate supplementation enhanced DNA methylation through a Sp1/Sp3-mediated transcriptional upregulation of genes coding for 2 DNA methyltransferases: DNMT3A and DNMT3B. In particular, Sp3 acted as an activator/enhancer of the DNMT3A and DNMT3B gene expression.	Folate supplementation enhanced DNA methylation with ↑ genes coding for 2 DNMT3A and DNMT3B	Vegetables (especially dark green leafy vegetables), fruits and fruit juices, nuts, beans, peas, seafood, eggs, dairy products, meat, grains, spinach, asparagus and brussels sprouts. Folate is available as a dietary supplement
[72]	GE	In vitro*:* Breast precancerous MCF10AT cells and cancer MCF-7 cells	Treatment with 50 μM GE (MCF10AT) and 100 μM GE (MCF-7 cells) for 3 d	Western blot to analyze DNMT protein expression	—	↓ Expression of DNMT1, DNMT3A, and DNMT3B, at 2 and 3 d of treatment in a time-dependent manner in MCF-7 cells ↓ Expression of DNMT1, but ↔ DNMT3A and DNMT3B, were found in MCF10AT cells	Soybeans
[73]	Parthenolide	In vitro: MV4-11 and Kasumi-1 AML cells	Treatment with 3.5 and 10 μM parthenolide	DNMT1 activity Assay kit (Epigentek) Immunoblotting and chemiluminescent detection kit (Pierce) to analyze DNMT1 protein levels qRT-PCR to analyze *DNMT1* gene expression	The parthenolide inhibits DNMT1 through both alkylation of the proximal thiolate of Cys1226 of the catalytic domain by its γ-methylene lactone and its association with subG1 cell cycle arrest or the interruption of transcriptional factor Sp1 binding to the promoter of *DNMT1*.	↓ DNMT1 activity by EC_50_ of 3.5 μM parthenolide DNMT1 protein level was totally depleted in MV4-11 cells and ↓ by 50% in Kasumi-1 cells when exposed to 10 μM ↓ Transcriptional level of DNMT1 to 20% of its control level, in MV4-11 cells treated with 10 μM	*Tanacetum parthenium*, known as feverfew
[74]	GE	In vitro*:* Human renal carcinoma A498, ACHN, and HEK-293 cells	Treatment with 50 μM GE for 3 d	DNMT activity and DNMT1, DNMT3A, and DNMT3B proteins measured using EpiQuik Assay kit (Epigentek)	—	↓ Downregulation of DNMT activity by treatment with 50 μM GE in all cell lines ↓ DNMT3B protein levels in all cell lines ↓ DNMT1 and DNMT3A protein levels in all cell lines except in A498 cells	Soybeans
[75]	EGCG, GE, myricetin and quercetin, hesperetin and naringenin, API and luteolin, garcinol, CU, and hydroxycinnamic acid	In vitro: Human esophageal (KYSE 150) and prostate (LNCaP and PC3) cancer cells	Treatment with 20 μmol/L EGCG and 20–50 μmol/L other polyphenolic compounds	Kinetic studies on the inhibition of DNMT	The interaction between EGCG and DNMT revealed a binding region with hemimethylated DNA and an active pocket for subsequent methylation. Catechol polyphenols may indirectly inhibit DNMT by generating SAH on their methylation by SAM.	↓ DNMT activities by all compounds at 20 and 50 μmol/L, but their activities were ↓ than those of EGCG GE was a weaker DNMT inhibitor than EGCG At 50 μmol/L, hydroxycinnamic acid, garcinol, and luteolin inhibited DNMT activity by >50%	EGCG: green tea GE: soybean
[76]	Annurca polyphenol extract	In vitro: Human colorectal cancer RKO, SW48, and SW480 cell lines	Treatment with 2 μM Annurca polyphenol extract	qRT-PCR to analyze DNMT1 and DNMT3B mRNA levels Western blot to analyze DNMT1 and DNMT3B protein expression	—	↔ Transcript levels between treated and control cells, whereas a ↓ was observed in protein expression 48 h after the end of the treatment for both DNMT1 and DNMT3B These results suggest that extract induces demethylation through posttranslational inhibition of both DNMT1 and DNMT3B.	Annurca apple flesh
[77]	Mahanine	In vitro: Prostate cancer PC3 and LNCaP cells.	Treatment with 1, 2, and 3 μg/mL mahanine for 3 d	DNMT activity Assay kit (Epigentek)	—	↓ DNMT activity in a dose-dependent manner after mahanine treatment (1, 2, and 3 μg/ml) for 3 d	*Micromelum minutum*
[78]	Caffeic acid and chlorogenic acid	In vitro: human DNMT	Treatment with IC_50_ values of caffeic acid and chlorogenic acid were 2.3 and 0.9 μM, respectively	Assay of enzymatic DNMT1 in vitro	Caffeic acid or chlorogenic acid inhibited DNA methylation through a noncompetitive mechanism largely because of the increased formation of SAH, a potent inhibitor of DNA methylation, resulting from COMT-mediated *O*-methylation of these dietary catechols.	Caffeic acid and chlorogenic acid (at 5 or 20 μM) exerted a very weak direct inhibition of DNMT1 in the absence of COMT In the presence of COMT, DNMT1 was strongly inhibited by caffeic acid and chlorogenic acid in a concentration-dependent manner	Coffee
[79]	EGCG	In vitro: Human cancer cell lines representing lymphoid (TK6 and Jurkat), myeloid (KG-1), or colorectal (HCT116)	Treatment with 10, 20, 20, 10 μmol/L EGCG in TK6, Jurkat, KG-1, and HCT116	DNMT activity assay	—	↔ EGCG failed to induce significant effects on DNA methylation	Soybeans
[80]	GE, Biochanin A, and Daidzen	In vitro: Human esophageal squamous carcinoma KYSE-510 cell lines.	KYSE-510 cells were treated with 1, 2, 5, 10, and 20 μmol/L GE for 6 d KYSE-510 cells were treated with nuclear GE or biochanin A or daidzein (5, 10, 20, 50, or 100 μmol/L) in 40 μL total volume for 6 d	qRT-PCR to analyze the expression levels of DNMT1, DNMT3A, DNMT3B DNMT activity Assay kit (Epigentek)	GE inhibits DNMT activity in a substrate- and methyl donor–dependent manner.	↔ mRNA expression levels of DNMT1, DNMT3A, DNMT3B after GE treatment (1, 2, 5, 10, and 20 μmol/L) for 6 d ↓ DNMT activity in a dose-dependent manner (20, 50, 100 μmol/L GE) in KYSE-510 cells Biochanin A and daidzein were less effective in inhibiting DNA DNMT activity	Soybeans
[81]	Tea polyphenols (catechin, epicatechin, and EGCG) and bioflavonoids (quercetin, fisetin, and myricetin)	Prokaryotic bacterial SssI DNMT and human DNMT1	Treatment with concentrations of catechin, epicatechin, and various flavonoids ranged from 1.0 to 8.4 μM (IC_50_ values), and EGCG concentration ranged from 0.21 to 0.47 μM (IC_50_)	Assay of enzymatic DNA methylation in vitro	Inhibition of DNA methylation by some DNMT by dietary polyphenols (catechin and epicatechin): one is the direct inhibition of the DNMTs (independent of COMT-mediated methylation), and the other is the indirect inhibition through increased formation of SAH during COMT-mediated methylation of the dietary chemicals. The strong inhibitory effect of EGCG on DNMT1 is attributable to its strong, direct inhibition of this enzyme, and the inhibition is greatly enhanced by Mg^2+^. The direct inhibition by quercetin or fisetin (at 20 μM) in the absence of COMT was rather weak, whereas myricetin exerted quite a strong direct inhibition.	Inhibition by tea catechins: IC_50_ values of catechin and epicatechin for ↓ of DNMT1 were 4.6 and 8.4 μM, respectively, with their maximal inhibition (∼80%) at the highest 20 μM inhibitor concentration ↓ DNMT1 activity 10- to 20-fold higher for EGCG (IC_50_ = 0.47 μM) than catechin and epicatechin Inhibition by bioflavonoids: ↓ DNMT1 activity by quercetin, fisetin, and myricetin with IC_50_ values of 1.6, 3.5, and 1.2 μM, respectively	Tea
[82]	Folate	In vitro: Mouse fibroblast (NIH/3T3) and Chinese hamster ovary (CHO-K1) cells, and 2 human colon adenocarcinoma cell lines (HCT116 and Caco-2)	Treatment without (deficient) or with 2.3 μM folic acid (control). NIH/3T3 and CHO-K1 cells were harvested after 12 d of growth whereas HCT116 and Caco-2 cells were harvested after 20 d of growth	DNMT activity kit assay (Epigentek) Western blot to analyze DNMT1 and DNMT3A protein expression	Folate deficiency determines the lack of SAH-mediated DNMT inhibition.	↓ DNMT activity of 28% in the folate-deficient NIH/3T3 cells and Caco-2 colon cancer cells than the folate-sufficient cells ↔ DNMT activity between the folate-deficient and folate-sufficient CHO-K1 cells, as well as sufficient HCT116 colon cancer cells ↓ DNMT1 and DNMTA protein expression in the folate-deficient NIH/3T3 cells but also in HCT116 and Caco-2 cells than in the folate-sufficient cells	Vegetables (especially dark green leafy vegetables), fruits and fruit juices, nuts, beans, peas, seafood, eggs, dairy products, meat, poultry, and grains, spinach, liver, asparagus, and brussels sprouts.
[83]	SFN	In vitro: Human colon adenocarcinoma Caco-2 cell line	Treatment with 50 μmol/L SFN	qRT-PCR to analyze DNMT1 expression	—	↓ DNMT1 expression (1.8-fold)	Cruciferous vegetables: broccoli, BSp, and brussels sprouts
[84]	Peyssonenynes A and B	In vitro: DNMT enzyme	Treatment with 16 and 9 μM for both peyssonenynes A and B	DNMT activity analyzed using a homogeneous scintillation proximity assay	—	↓ Pure peyssonenynes A and B showed comparable activity (16 and 9 μM, respectively) in DNMT1 enzyme inhibition	Fijian red marine alga *Peyssonnelia caulifera*
[85]	Psammaplin A and bisaprasin, and psammaplin G	In vitro: Solid tumor cell (Colon38, ColonH116, Lung H125) and either leukemia cells (L1210 or CEM) or normal cells (CFU-GM)	Treatment with 18.6, 12.8, and 3.4 nM for Psammaplin A, G, and bisaprasin, the IC_50_ for each compound	DNMT activity enzymatic Assay kit	—	↓ Psammaplin A, bisaprasin, and psammaplin G are inhibitors of DNMT	*Pseudoceratina purpurea* marine sponge
**In vivo studies**							
[86]	*Espermaplus* supplement, containing liposoluble vitamins (A, D, E, and K) and water-soluble vitamins (B1, B2, B6, B12, and C; biotin; folic acid; nicotinic acid; pantothenic acid; and choline), ω-3 fatty acids, amino acids (lysine, methionine, threonine, and tryptophan), oligo-elements (zinc, selenium, copper, iron, manganese, and iodine)	In vivo: 30 boars 2 y old	Treatment with 75 g/d of *Espermaplus* supplement for 12 wk	qRT-PCR to quantify DNMT3A and DNMT3B expression	—	↔ No statistically significant differences between experimental and control groups in DNMT3A and DNMT3B expression	Vitamins: fish, eggs, dairy products, green vegetables, meat, legumes, whole grains, and fruit; ω-3 fatty acids: fatty fish, seeds, walnuts, and algae Essential amino acids: meat, eggs, dairy products, legumes, soy, whole grains Oligo-elements: red meat, whole grains, legumes, nuts, seeds, iodized salt, seaweed
[87]	Vitamin E	In vivo: C57BL/6J male mice	Treatment with a HFD or CD with and without vitamin E supplementation (4.5 mg/kg body weight) for 4 mo	qRT-PCR to analyze DNMT1 expression	—	In colon cells, no changes (↔) in DNMT1 expression in cells of HFD + vitamin E compared with HFD; whereas ↑ DNMT1 expression (87%) observed in CD + vitamin E compared with CD In the liver, ↓ DNMT1 expression in CD + vitamin E compared with CD (79%) and in HFD + vitamin E compared with HFD (68%)	Seeds and nuts: almonds, hazelnuts, peanuts, peanut butter Fish: abalone, trout, salmon Vegetables: red sweet pepper, turnip greens, butternut squash Fruit: mamey sapote, avocado, mango, kiwi fruit
[88]	EGCG	In vivo: C57BL/6J male mice	Treatment with HFD or CD with and without EGCG supplementation for 4 mo	qRT-PCR to analyze DNMT1 expression	Inhibitory mechanisms are on one hand via direct pathways and on the other hand indirect via DNMT-mediated DNA methylation through increased formation of SAH, a potent inhibitor of SAM-mediated reactions.	In colon cells, no changes (↔) in DNMT1 expression in CD + vitamin E compared with CD, whereas ↑ DNMT1 expression in HFD + vitamin E compared with HFD. In the liver, ↓ DNMT1 expression in CD + vitamin E compared with CD (75%), whereas no differences (↔) in HFD + vitamin E compared with HFD (68%)	Soybeans
[89]	GTP	In vivo: male severe combined immunodeficiency mice with androgen-dependent human LAPC4 prostate cancer cell subcutaneous xenografts	Green tea administered daily in drinking water for 13 wk. Composition of brewed green tea in milligrams per liter: EGC 202 ± 11, EGCG 397 ± 28, EC 52 ± 5, ECG 62 ± 5, and catechin 8 ± 2	qRT-PCR to quantify DNMT1 expression Western blot to analyze DNMT1 protein levels	—	↓ DNMT1 gene and protein expression significantly of 55% and 40%, respectively	Green tea
[90]	GE	In vivo: BALB/c nude mice	Treatment with 2 mg GE	qRT-PCR to analyze DNMT1, DNMT3A, DNMT3B expression	GE alters the configuration of chromatin and acts as a DNMT inhibitor.	↓ Expression of DNMT3B in GE treatment group ↔ No obvious changes in the DNMT1 and DNMT3A isoforms	Soy
[91]	EGCG	In vivo: 6–7 wk-old female SKH-1 hairless mice	Treatment with EGCG uniformly mixed in hydrophilic cream and topically applied (∼1 mg/cm^2^ skin area) onto the mouse skin 20–25 min before each UVB exposure	DNMT activity expressed as counts per minute per microgram of protein, quantifying the units of radioactivity present in each sample by using a liquid scintillation counter. DNMT protein content estimated using the DC protein assay kit (BioRad)	EGCG may influence the supply of methyl groups for the formation of SAM and/or modify utilization of methyl groups by processes including shifts in DNMT activity.	↓ DNMT1 activity by 41% in the UVB-irradiated skin compared with normal skin	Green tea
**Ex vivo studies**							
[92]	Evodiamine and BBR	Ex vivo: Colon tissues from neonatal rats aged 7 d	Treatment with BBR 10 μM or evodiamine 15 μM	qRT-PCR to analyze DNMT1, DNMT3A, and DNMT3B expression	BBR and evodiamine effect the expression of miR-29a. miR-152, miR-429, and miR-29a directly bind to the DNMT1, DNMT3A, and DNMT3B transcript, respectively, and posttranscriptionally mediate expression of those genes.	↓ Expression levels of DNMT1, DNMT3A, DNMT3B after a 24-h treatment with both BBR and evodiamine ↑ Expression levels of DNMT1, DNMT3A, DNMT3B after a 48-h treatment with both BBR and evodiamine ↓ Expression level of DNMT1 after 72 h of treatment ↑ Expression levels of DNMT3A and DNMT3B after 72h of treatment	BBR: Chinese herbal medicine *Coptis chinensis*. Evodiamine: *Evodia rutaecarpa*
[93]	Folate/methyl	Ex vivo: brain cortical tissue from weaning male F344 rats	Feeding a folate/methyl-deficient diet for 18 and 36 wk	Western blot to analyze DNMT1, DNMT3A, DNMT3B protein expression	—	↓ DNMT1 protein levels by ∼50% Protein level at 18 and 36 wk of deficiency was, respectively, 1.4 and 1.7 times ↑ than in brains of the age-matched control rats	Green leafy vegetables, legumes, citrus fruits, and dietary supplements
**Combined studies**							
[94]	SFN and BSp	In vitro: Breast cancer MDA-MB-157 and MDA-MB-231 cells In vivo: WT Her2/neu breast cancer mouse model	Treatment with 5 μM SFN for 72 h in vitro Treatment with 26% (w/w) BSp diet in vivo: -pregestational maternal BSp treatment (P-BSp): BSp diet to the mother from postnatal 3 wk of age until 10 wk -long-term maternal BSp treatment (L-BSp): diet to the mother from postnatal 3 wk of age until the weaning of their offspring (21 d of age) -postnatal adult BSp treatment (A-BSp): diet to female offspring from 10 wk of age until the termination of the experiment	qRT-PCR to analyze DNMT1 and DNMT3A expression ex vivo Western blot to analyze DNMT1 and DNMT3A protein levels ex vivo and DNMT3A and DNMT3B in vitro DNMT activity Assay Kit (Epigentek)	—	↓ Levels of DNMT1 and DNMT3A, as well as their protein levels, in both maternal BSp dietary exposures (P-BSp and L-BSp) ↓ Expression of DNMT1 at both transcript and protein levels after BSp diet in the adult group (A-BSp) ↔ DNMT3A gene expression after BSp diet ↓ Protein levels of DNMTs after SFN treatment in ERα-negative breast cancer cell lines ↔ DNMT activity in P-BSp, L-BSp, and A-BSp	Common cruciferous vegetables and BSp
[95]	Quercetin.	In vitro: AML cell lines (HL60 and U937) In vivo: 2 human xenograft AML models	Treatment 50 and 75 μmol/L for 48 or 72 h	Western blot to analyze protein levels qRT-PCR to quantify DNMT1 and DNMT3A expression	Quercetin decreases STAT3, a transcription factor that also regulates DNMT, at the protein and at the message level and p-STAT3 at the protein level. Quercetin inhibits DNMT1 and DNMT3A expression in a STAT3-dependent manner.	↓ DNMT1 and DNMT3A expression, at the protein and message levels, in both in vitro and in human xenograft models in a dose- and time-dependent manner	Vegetables, fruits, nuts, tea, red wine, and propolis
[96]	BBR	In vitro: NSCLC cells (A549 and H1975) Ex vivo: lung cancer cells from female nude mice	Treatment with 6.25, 12.5, 25, 50, 100 μM for 24 h in cells Treatment with 10 mg/kg in mice	qRT-PCR to measure expression of DNMT1 in vitro Western blot to evaluate DNMT1 protein in vitro and ex vivo	BBR inhibits the growth of NSCLC cells through inhibition of Sp1 and PDPK1; this results in the reduction of DNMT1 gene expression. The interplay of PDPK1 and Sp1 contributes to the inhibition of DNMT1 in response to BBR.	↓ Protein and expression of DNMT1 in H1975 and A549, in a dose-dependent manner ↓ DNMT1 protein expression in vivo	Rhizome
[97]	EGCG, GTPs, and SFN in BSp	In vitro: Breast cancer MCF-7, MDA-MB-231, and MDA-MB-157 cells In vivo: mouse orthotopic xenografts	In vitro: treatment with 20 μM EGCG and 10 μM SFN for a total of 3 d In vivo: mice were fed with both 0.3% GTPs and 13% BSp diets for 2 wk before injection and mice continued to receive the experimental diet throughout the study	qRT-PCR to analyze DNMT1 expression Western blot to analyze DNMT1 protein levels DNMT activity Assay Kit (Epigentek)	—	↓ DNMT1 mRNA and protein levels after combination treatment with EGCG and SFN in both of the tested cell lines ↓ DNMT enzymatic activities after combination treatment ↓ Expression of DNMT1 in mouse orthotopic xenografts after dietary combination treatment with GTPs and BSp	Green tea and BSp
[98]	CU	In vitro: leukemia cell lines, K562 (erythroleukemic cell line), MV4–11, and HL-60 (AML) In vivo: Female athymic nu/nu mice (4–6 wk old, 18–22 g) Ex vivo: mononuclear cells isolated from bone marrow of mice with AML	Treatment with CU at various concentrations (5, 10, 20 μM) for 24, 48 and 72 h. Mice were randomly assigned into 2 cohorts and CU was given intraperitoneally as a solution of DMSO at a dose of 100 mg/kg daily, 5 d/wk, for 4 weeks. The placebo formulation was used as a control	RT–qPCR to analyze DNMT1. Immunoblotting for DNMT1 protein expression level	CU downregulates DNMT1 expression in AML cells, at least in part, through downmodulation of the Sp1 and the NF-κB component, p65, which physically interact and bind to the *DNMT1* promoter. CU can cause hypomethylation via blockade of the catalytic thiol group of DNMT1. CU acting as both a chemical inhibitor and a transcriptional modulator of DNMT1.	↓ DNMT1 expression in AML cell lines, both in vitro and in vivo, and in primary AML cells ex vivo ↓ DNMT1 protein level in a dose-dependent fashion	Turmeric

Information extracted from the selected studies: author and year of publication, dietary molecule tested, experimental models adopted (in vitro, in vivo, ex vivo, combined studies), dose of food molecule tested, methods used, mechanism of action, main results, and class and major source. Legend: ↓ Decreased; ↑ Increased; ↔ Unchanged.

deoxycytidine; 6-PN, 6-prenylnaringenin; Ado, Adenosine; AhR, aryl hydrocarbon receptor; Akt, protein kinase B; AML, acute myeloid leukemia; AMPKα, AMP-activated protein kinase α; API, apigenin; BBR, berberine; BPH-1, benign prostate hyperplasia; BRCA-1, breast cancer type 1 susceptibility protein; BSp, broccoli sprouts; CD, control diet; ChIP, chromatin immunoprecipitation; COMT, catechol-*O*-methyltransferase; CU, curcumin; DL-Hcy, DL-homocysteine; DNMT, DNA methyltransferase; DS, dioscin; E2, 7β-estradiol; EC, (–)-epicatechin; ECG, (–)-epicatechin-3-gallate; EGC, (–)-epigallocatechin; EGCG, epigallocatechin-3-gallate; Erα, estrogen receptor alpha; ERK1/2, extracellular signal regulated kinase; EZH2, enhancer of zeste homolog 2; FQ-PCR, fluorescent quantitative PCR; GA, gallic acid; GE, genistein; GSP, grape seed proanthocyanidin; GTP, green tea polyphenol; HDAC, histone deacetylase; HFD, high-fat diet; Lig, Z-ligustilide; NaB, sodium butyrate; NF-κB (p65), nuclear factor-kappa B (p65 subunit); NSCLC, non-small cell lung cancer; PDPK1, 3-phosphoinositide-dependent protein kinase-1; PEITC, phenethyl isothiocyanate; PepE, peperomin E; PI3K, phosphatidylinositol 3-kinase; RAS, Radix Angelicae Sinensis; qRT-PCR, quantitative reverse transcriptase-polymerase chain reaction; SAH, S-denosylhomocysteine; SAM, S-denosylmethionine; SFN, sulforaphane; Sp1, specificity protein 1; Sp3, specificity protein 3; SRA, SET and RING-associated domain; STAT3, signal transducer and activator of transcription 3; TCDD, 2,3,7,8-tetrachlorobenzo(p)dioxin; TCS, trichosanthin; TNF, tumor necrosis factor; TQ, thymoquinone; UHRF1, ubiquitin like with PHD and ring finger domains 1; WYRE, wild yam root extract; WNT, wingless-related integration site; WT, wild type.

### Selection criteria and literature screening process to identify natural dietary molecules and extracts affecting DNMT

Screening and filtering were carried out, and relevant articles were selected considering the following inclusion criteria chosen for this systematic review. Articles investigating the effects of bioactive compounds naturally occurring in food sources on DNMTs, even when administered in purified or synthetic form, were included, whereas studies assessing exclusively synthetic, non–food-derived molecules were excluded. Further inclusion criteria were applied: original articles (not reviews, editorials, brief communications, or conference abstracts) and articles in the English language. Furthermore, cited papers in the selected publications and the reference lists of relevant reviews during the screening process were scanned and also considered. For each included study, all results compatible with the outcome domain of DNMT modulation were systematically sought across all reported measures, time points, and analyses. When multiple experimental conditions were available, data extraction prioritized the most comprehensive and relevant results directly related to the outcome domain. When necessary, data were extracted or converted to ensure consistency across studies; for example, concentrations reported in different units were standardized to micromolar or millimolar concentrations to allow comparison. Risk of bias was assessed considering reproducibility, control adequacy, sources of variability, and methodological transparency in preclinical studies. Two reviewers independently conducted the assessment and data collection, resolving discrepancies by consensus or involving a third reviewer. Non–peer-reviewed studies or articles without available full texts or without experimental data on DNMT were excluded.

Given the large volume and methodological variability of the included studies, we classified the 76 studies according to the type of food-derived bioactive compound investigated: bioactive dietary molecules, natural extracts, and multicomponent compounds (Table 2), providing a practical framework to facilitate cross-comparison and deepen insight into structure–function relationships relevant to DNMT modulation. Briefly, the term “bioactive dietary molecules” refers to isolated compounds naturally occurring in foods (e.g., EGCG, CU, GE), typically studied as single agents. “Natural extracts” denote complex mixtures derived from plants or fruits, often containing multiple constituents with potential synergistic effects. Finally, studies of “multicomponent compounds” intentionally tested 2 or more bioactive molecules or extracts together to assess potential synergism in DNMT inhibition or modulation. This categorization was developed to systematize the growing diversity of bioactive compounds with reported DNMT-modulating properties and to enable a more structured evaluation of their mechanisms of action, experimental contexts, and potency, as described in **Supplemental Results**.

**TABLE 2:** Food-derived bioactive compounds with epigenetic effects on DNMT: classification into bioactive dietary molecules, extracts, and multicomponent compounds (multiple dietary molecules, multiple extracts, or extracts and dietary molecules) including study count and study type.

References	Compound	Total number (*n*)of studies	Study types
**BIOACTIVE DIETARY MOLECULES**			
[50]	Apigenin	*n* = 1	*n* = 1 in vitro
[26,96,92]	Berberine	*n* = 3	*n* = 2 in vitro *n* = 1 combined (in vitro and ex vivo from animals)
[66,98]	Curcumin	*n* = 2	*n* = 1 in vitro *n* = 1 combined (in vitro, in vivo animal model, and ex vivo from animals)
[36]	Dioscin	*n* = 1	*n* = 1 in vitro
[42,46,64,65,67,69,79,88,91]	Epigallocatechin-3-gallate	*n* = 9	*n* = 7 in vitro *n* = 2 in vivo animal model
[39]	Emodin	*n* = 1	*n* = 1 in vitro
[71,82,93]	Folate	*n* = 3	*n* = 2 in vitro *n* = 1 ex vivo from animals
[33,49]	Gallic acid	*n* = 2	*n* = 2 in vitro
[16,31,53,80,72,74,90]	Genistein	*n* = 7	*n* = 6 in vitro *n* = 1 in vivo animal model
[54,77]	Mahanine	*n* = 2	*n* = 2 in vitro
[73]	Parthenolide	*n* = 1	*n* = 1 in vitro
[40]	Peperomin E	*n* = 1	*n* = 1 in vitro
[37]	Phenethyl isothiocyanate	*n* = 1	*n* = 1 in vitro
[95,32,44]	Quercetin	*n* = 3	*n* = 2 in vitro *n* = 1 combined (in vitro and in vivo animal model)
[38,41,70]	Resveratrol	*n* = 3	*n* = 3 in vitro
[15,35,43,45,52,58,59,63,83,94]	Sulforaphane	*n* = 10	*n* = 9 in vitro *n* = 1 combined (in vitro and in vivo animal model)
[60]	Trichosanthin	*n* = 1	*n* = 1 in vitro
[34,87]	Vitamin E	*n* = 2	*n* = 1 in vitro *n* = 1 in vivo animal model
[48]	β-elemene	*n* = 1	*n* = 1 in vitro
**NATURAL EXTRACTS**			
[76]	Annurca apple polyphenols	*n* = 1	*n* = 1 in vitro
[27]	Black seed oil and its active compound thymoquinone	*n* = 1	*n* = 1 in vitro
[94]	Broccoli sprouts	*n* = 1	*n* = 1 combined (in vitro and in vivo animal model)
[62]	Grape seed proanthocyanidins	*n* = 1	*n* = 1 in vitro
[69,89]	Green tea polyphenols	*n* = 2	*n* = 1 in vitro *n* = 1 in vivo animal model
[29]	Hop extract and its bioactive compound 6-PN	*n* = 1	*n* = 1 in vitro
[30]	Methanolic extract of *Paederia foetida* leaves	*n* = 1	*n* = 1 in vitro
[57]	RAS and its active component ligand	*n* = 1	*n* = 1 in vitro
[81]	Tea polyphenols (catechin, epicatechin, EGCG) and bioflavonoids (quercetin, fisetin, myricetin)	*n* = 1	*n* = 1 in vitro
[36]	Wild yam root extract	*n* = 1	*n* = 1 in vitro
**MULTICOMPONENT COMPOUNDS**			
**a) Multiple dietary molecules**			
[78]	Caffeic acid + chlorogenic acid	*n* = 1	*n* = 1 in vitro
[51]	EGCG + NaB	*n* = 1	*n* = 1 in vitro
[47]	EGCG + procyanidin B2	*n* = 1	*n* = 1 in vitro
[55,97]	EGCG + SFN	*n* = 2	*n* = 2 in vitro
[84]	Peyssonenynes A + B	*n* = 1	*n* = 1 in vitro
[38,41]	Resveratrol + pterostilbene	*n* = 2	*n* = 2 in vitro
[80]	GE + daidzein + biochanin A	*n* = 1	*n* = 1 in vitro
[85]	Psammaplin A + bisaprasin + psammaplin G	*n* = 1	*n* = 1 in vitro
[28]	SFN + GE + NaB	*n* = 1	*n* = 1 in vitro
[56]	EGCG + GE + CU + resveratrol + withaferin A + guggulsterone	*n* = 1	*n* = 1 in vitro
[68]	Baicalein + myricetin + protocatechuic acid + phloretin + sinapic acid + syringic acid + resveratrol + rosmarinic acid + ellagic acid + betanin + cyanidin + galangin	*n* = 1	*n* = 1 in vitro
[75]	EGCG + GE + myricetin + quercetin + hesperetin + naringenin + API + luteolin + garcinol + CU + hydroxycinnamic acid	*n* = 1	*n* = 1 in vitro
[86]	Espermaplus supplement (vitamins, ω-3 fatty acids, amino acids, and oligo-elements)	*n* = 1	*n* = 1 in vivo animal model
**b) Extract and dietary molecule**			
[61]	GTPs + SFN	*n* = 1	*n* = 1 in vitro
**c) Multiple extracts**			
[97]	GTPs + Broccoli sprouts	*n* = 1	*n* = 1 in vivo animal model

Abbreviations: 6-PN, 6-prenylnaringenin; API, apigenin; CU, curcumin; EGCG, epigallocatechin-3-gallate; GE, genistein; GTP, green tea polyphenol; NaB, sodium butyrate; RAS= Radix Angelicae Sinensis; SFN, sulforaphane.

### Determination of minimum effective dose for induction of DNMT inhibition

To determine the minimum effective dose of each dietary molecule that can inhibit DNMT activity, data were extracted using the following steps:

1) Dose–response data collection from studies reporting DNMT inhibition at various concentrations of each food-derived bioactive compound were systematically reviewed.
2) The effective concentration of each food-derived bioactive compound was analyzed to identify the minimum concentrations that show significant DNMT inhibition.

## Results

### Effect of food-derived compounds on DNMT modulation

Table 1 provides a detailed overview of the 76 studies selected for this review, which investigated the effects of food-derived and bioactive dietary molecules on DNMT modulation across various experimental models. The vast majority (*n* = 63; 82.9%) were in vitro studies, mainly employing cancer cell lines from the following tissues: breast (*n* = 20; 31.7%), prostate (*n* = 11; 17.5%), colon (*n* = 10; 15.9%), cervix (*n* = 6; 9.5%), and lung (*n* = 6; 9.5%), and epithelial (*n* = 4; 6.3%) and other tissues, including those of pancreatic, skin, neuronal, ovarian, endothelial (cardiovascular and cerebrovascular), hematological, renal, and esophageal origin. A smaller subset (*n* = 3; 4.8%) focused on DNMT enzymatic activity using either human enzymes or prokaryotic SssI DNMTs. Six studies (7.9%) were conducted in vivo and 2 ex vivo (2.6%). A few studies (*n* = 5; 6.6%) adopted combined approaches integrating in vitro data with in vivo or ex vivo systems. No clinical trials or epidemiological studies were identified.

Various methodologies were employed across the studies. qRT-PCR (*n* = 54; 71.1%) and western blotting (*n* = 43; 56.6%) were used to assess DNMT expression and protein levels, respectively. DNMT activity assays were applied in 23 studies (30.3%), whereas ELISA (*n* = 6; 7.9%) and immunofluorescence (*n* = 4; 5.3%) were less commonly used. A subset of studies employed molecular docking and structural modeling predicting how compounds such as EGCG, CU, GE, sulforaphane (SFN), parthenolide, thymoquinone (TQ), and peperomin E (PepE) may bind to conserved catalytic or substrate recognition domains of DNMT1 and DNMT3B [27,40,42,43,53,73,81,99]. Furthermore, few studies integrated multiple levels of analysis—such as combining expression, activity, and functional methylation readouts—within the same experimental design [28,40,53,[60], [61], [62],^65^,^69^,^94^,^97^].

As detailed in Table 1, the vast majority of the included studies (89.5%) reported a downregulation or suppression of DNMT enzymatic activity, mRNA expression, or protein levels, highlighting a predominant inhibitory trend across different compounds and experimental models through direct and indirect mechanisms. Direct inhibition was substantiated by a subset of studies (4.8%) that employed enzymatic assays with recombinant DNMTs or bacterial analogs (e.g., SssI), demonstrating a measurable reduction in DNMT activity [81,84,85]. Molecular docking and structural modeling studies provided compelling evidence for the ability of specific dietary compounds to bind directly to conserved catalytic or substrate-binding residues of DNMT isoforms [27,40,42,53,73,81,99]. These studies showed EGCG forms multiple hydrogen bonds with catalytic residues of DNMT1—such as Glu1265, Arg1310, Arg1311, Lys1482, Arg832, and Arg823—obstructing the access of both the methyl donor and cytosine substrate [42,81]. Similarly, CU covalently binds to the thiolate group of Cys1226 in DNMT1, irreversibly inhibiting its catalytic activity [99]. Parthenolide exerts its inhibitory effects via alkylation at the same residue through its γ-methylene lactone moiety [73]. GE interacts with Phe1145 and Leu1153, residues involved in stabilizing cytosine within the DNMT1 active site, alongside Pro1225 and Val1580 [53]. SFN, on the other hand, binds within the substrate-binding cavity of DNMT3B and interacts with residues such as Phe581, Asp582, Cys651, and Trp834 [43]. TQ forms hydrogen bonds with Lys697 and Lys1242 [27], whereas PepE interacts with Gly1147, Glu1168, and Phe1145 in the DNMT1 catalytic domain [40].

Indirect mechanisms were also frequently reported (see Table 1), including alterations in transcription factor expression (e.g., Sp1 by berberine [BBR], CU, and parthenolide [73,98,96]; STAT3 by quercetin [95]; NF-κB by CU and PepE [40,98]), disruption of methyl donor availability (e.g., folate, resveratrol [70,82]), and post-translational regulation. Notably, EGCG interferes with DNMT3A protein stability by reducing its interaction with the E3 ubiquitin ligase UHRF1, promoting its degradation [46]. Other compounds such as mahanine and EGCG also induce DNMT degradation via the ubiquitin–proteasome pathway [46,54]. Additional pathways include miRNA-mediated regulation (e.g., BBR and evodiamine [92]) and chromatin remodeling interactions, as reported for SFN and resveratrol [70,58].

Certain bioactive compounds—particularly BBR [26]—change microbial composition, leading to the production of microbiota-derived metabolites, such as butyrate, which can act as an endogenous effector by modulating DNMTs and thereby influencing DNA methylation patterns. Short-chain fatty acids (SCFAs) such as sodium butyrate (NaB), which is produced via microbial fermentation, reduce DNMT expression and activity, particularly when combined with other bioactive compounds, including GE, SFN, or EGCG [28,97,51].

DNMT inhibition was frequently (31.6% of the studies) shown to be dose- and time-dependent, with more pronounced effects at higher concentrations and longer exposure durations, as reported by multiple in vitro studies [27,43,53,40,60,96,95,46,54,30,32,48]. In particular, EGCG and GE [42,67,80] exerted measurable effects within the 50 to 150 μM and 60 to 100 μM ranges, respectively. Time dependence was also a critical factor, with enhanced DNMT inhibition observed after extended exposure—most commonly between 24 and 72 h [43,95,46].

In 16 studies (20.8%), treatments combining compounds (for example, SFN + GE + NaB, EGCG + SFN, and other combinations) showed stronger inhibitory effects than individual compounds alone [51,97,28,61,55].

Some studies were identified as being at higher risk of bias due to incomplete methodological descriptions (6.6%) or insufficient investigation of mechanisms of action (47.4%), as detailed in Supplemental Table 2.

### Classification of food-derived bioactive compounds and experimental models

Table 2 summarizes the 76 studies reviewed, grouping the investigated food-derived bioactive compounds into bioactive dietary molecules, natural extracts, and multicomponent formulations. Bioactive dietary molecules were the most studied (*n* = 55; 72.4%), followed by natural extracts (*n* = 11; 14.5%) and multicomponent compounds (*n* = 17; 22.4%). Five studies explored overlapping categories. Among bioactive dietary molecules, SFN, EGCG, and GE were the most frequently studied.

### MECs of bioactive compounds

Table 3 summarizes the MECs reported for DNMT inhibition by bioactive dietary molecules and natural extracts in preclinical studies. Among bioactive dietary molecules, SFN and mahanine emerged as the most potent DNMT inhibitors, with MECs as low as 1 to 5 μM and 2.9 to 8.6 μM, respectively [43,54]. Other compounds demonstrating consistent activity at relatively low concentrations include EGCG, CU, resveratrol, and gallic acid, which are typically effective at concentrations of 5 to 25 μM, indicating a moderate potency across a wide range of models and conditions [42,98,70,80,33]. By contrast, BBR exhibited a broader and less predictable effective range, likely reflecting differences between studies in cell type, exposure duration, and intracellular bioavailability [92,26]. Among natural extracts, Annurca apple polyphenols demonstrated the strongest DNMT inhibition at a concentration of 2 μM [76], comparable to that of potent single molecules. Green tea polyphenols (GTPs) and hop extracts also showed notable activity [69,29]. Conversely, extracts such as black seed oil and Radix Angelicae Sinensis required significantly higher concentrations, reflecting lower potency likely due to their complex composition and lower content of active constituents [27,57]. Multicomponent compounds were excluded from the MEC analysis because of their synergistic nature, which made it challenging to attribute effects to individual components.

**TABLE 3:** Minimum effective dose of the main dietary DNMT inhibitors.

References	Compound/extract	Minimum effective concentration	Experimental model	Tissue/cell type
**Dietary molecules**				
[96]	Berberine	6.25–100 μM	In vitro	NSCLC (A549 and H1975)
[98]	Curcumin	5–20 μM	In vitro and ex vivo	In vitro: K562 (erythroleukemic cell line), MV4–11, and HL-60 (AML) Ex vivo: primary AML cells
[42,67]	Epigallocatechin-3-gallate	10–25 μM	In vitro	HeLa cells Jurkat T cells
[33]	Gallic acid	10 μM	In vitro	NSCLC (A549 and H1975)
[80]	Genistein	20–50 μM	In vitro	KYSE 510 cells
[77]	Mahanine	2.9–8.6 μM	In vitro	PC3 and LNCaP cells
[32]	Quercetin	25–50 μM	In vitro	HeLa cells
[70]	Resveratrol	5–20 μM	In vitro	MCF-7 cells
[58,52]	Sulforaphane	1–5 μM	In vitro	TRAMP C1 cells JB6 P+ cells
**Natural extracts**				
[76]	Annurca apple polyphenols	2 μM	In vitro	RKO, SW48, and SW480 cell lines
[27]	Black seed oil	0.6%–1.25%	In vitro	Jurkat, MCF-7, and HeLa cells
[69]	Green tea polyphenols	1–20 μg/mL	In vitro	LNCaP cells
[29]	Hop extract and its bioactive compound (6-PN)	5 μg/mL (hop extract) 1 μM (6-PN)	In vitro	MCF-7 cells
[57]	Radix Angelicae Sinensis	58.5 μg/mL	In vitro	TRAMP C1 cells

Abbreviations: 6-PN, 6-prenylnaringenin; AML, acute myeloid leukemia; NSCLC, non-small cell lung cancer.

A detailed summary and comprehensive description of the study-specific findings—including molecular targets, experimental conditions, and outcomes related to DNMT modulation—are provided in the Supplemental Results.

## Discussion

### Translational perspectives on DNMT-targeting nutrients

This systematic review of the available literature is the first to provide early evidence of the DMNT-modulatory activity of food-derived bioactive compounds. A total of 76 studies met the inclusion criteria, with the vast majority (82.9%) conducted *in vitro*, primarily on cancer cell lines from breast, prostate, colon, cervix, lung, and other epithelial or organ-specific tissues. These studies consistently show that various dietary molecules—administered as isolated compounds, in synergistic combinations, or within complex natural extracts—can influence DNMT expression and activity through mechanisms such as direct enzymatic inhibition, transcriptional regulation, or signal transduction pathways.

Although these preclinical findings provide a solid mechanistic foundation, the translational trajectory toward clinical application is still in its early phase. Only a limited number of studies have progressed beyond cell-based models; 6 were conducted *in vivo*, 2 *ex vivo,* and 5 using integrated approaches combining various biological levels of investigation. Crucially, no clinical trial to date has been designed specifically to evaluate the DNMT-inhibitory effects of nutritional interventions. This gap likely reflects the inherent challenges in translating epigenetic modulation to human settings, including the need for long-term follow-up, dietary standardization, and the identification of robust, sensitive biomarkers. However, the absence of DNMT-specific trials should not be interpreted as a lack of translational progress. Recent human intervention studies have successfully demonstrated that epigenetic changes induced by diet and supplementation can be measured in humans, particularly through the use of epigenetic clocks—innovative tools that estimate biological age by analyzing specific DNA methylation patterns. Our own research exemplifies this translational direction [23]. After characterizing the antioxidant and anti-aging effects of *M. didyma L.* extract in vitro, we conducted a randomized clinical trial in aging workers. The intervention resulted in a stabilization of DNAmAge, as assessed by epigenetic clock analysis, accompanied by increased telomere length, higher mitochondrial DNA copy number, and measurable improvements in biological function and quality of life. Similarly, the DO-HEALTH study [24] showed that combined supplementation with vitamin D, ω-3 fatty acids, and physical activity significantly impacted DNAmAge in older adults. Together, these findings validate the feasibility and clinical relevance of using epigenetic age as a dynamic biomarker to assess the effects of nutriepigenetic interventions and support the development of personalized, preventive strategies targeting biological aging.

### Experimental techniques and structural models for investigating DNMT modulation

Studies included in this review employed robust experimental approaches to assess the modulation of DNMTs. qRT-PCR and western blotting were the most frequently used techniques to evaluate gene and protein expression levels, providing reliable molecular evidence of DNMT regulation. Nearly 30% of studies complemented expression data with DNMT activity assays, which are critical to confirm that changes in transcriptional or translational levels correspond to functional enzymatic modulation. Although used less frequently, ELISA and immunofluorescence assays contributed additional layers of validation in selected studies, particularly in confirming protein localization and quantification.

Moreover, a subset of studies employed molecular docking and structural modeling to provide mechanistic insights into the interaction between food-derived bioactive compounds and DNMT enzymes [27,40,42,43,53,73,81,99]. These modeling studies, although not focused on functional outcomes, strengthen the mechanistic plausibility of direct DNMT inhibition and provide a useful framework for prioritizing compounds for further validation.

Only a few studies integrated multiple levels of analysis, combining gene/protein expression data with enzymatic activity measurements and functional methylation outcomes within the same experimental design. This gap underscores the need for more integrative methodological frameworks capable of linking molecular effects to functional epigenetic and phenotypic outcomes. As the field progresses toward clinical applications, harmonizing methods and adopting multidimensional approaches will be essential for strengthening causal inference and comparability across studies.

### Mechanisms of DNMT inhibition by dietary bioactives: direct binding, indirect modulation, dose–time dependency, microbiome interactions, and synergistic strategies

A key discovery of this review is the consistent evidence supporting the inhibitory effects of food-derived bioactive compounds on DNMTs through direct and indirect mechanisms, with significant implications for epigenetic regulation.

Direct inhibition occurs through covalent binding to catalytic residues (e.g., Cys1226 in DNMT1 by CU and parthenolide) or through hydrogen bonding within substrate-binding domains (e.g., Lys697, Glu1168, Phe1145) as observed for several compounds—EGCG [42,81], CU [99], parthenolide [99], GE [53], SFN [43], TQ [27], and PepE [40]. This suggests that DNMTs share specific structural features that make them susceptible to inhibition by certain food-derived bioactive compounds. Although these findings come mainly from preclinical models, they offer a credible mechanistic explanation for the frequent reports of DNMT inhibition and support the potential use of dietary or supplemental compounds as epigenetic modulators in future preventive and therapeutic approaches.

Indirect mechanisms —transcription factor modulation (e.g., Sp1 [73,98,96], STAT3 [95], NF-κB [40,98]), interference with protein stability via ubiquitin–proteasome degradation [94,84], and miRNA-mediated post-transcriptional regulation [92] and chromatin remodeling interactions [70,58]—were also frequently reported, revealing a broader landscape through which dietary components can influence DNA methylation beyond direct catalytic inhibition. Together, these mechanisms illustrate the multifaceted and pleiotropic nature of dietary DNMT inhibitors, which act not only on the enzyme itself but across upstream and downstream regulatory networks.

Approximately 31.6% of the studies analyzed in this review reported a clear dose- and/or time-dependent inhibition of DNMT activity, highlighting a pharmacodynamic profile consistent with specific target engagement. For example, EGCG and GE [42,67,80] showed measurable effects within the 50 to 150 μM and 60 to 100 μM ranges, respectively. These concentrations, which exceed those achievable through standard diets, could be reached through carefully designed nutraceutical formulations or controlled dietary protocols tailored for preventive interventions. The temporal dimension—most commonly between 24 and 72 h [43,95,46]—was also a critical factor, reinforcing the concept of cumulative epigenetic modulation and suggesting that both intensity and duration of exposure are key determinants of biological efficacy. Together, these pharmacodynamic characteristics support the plausibility of DNMTs as responsive epigenetic targets and provide a foundation for optimizing dosage strategies in future translational and clinical applications.

Beyond absolute intake, several factors influence the bioavailability and metabolic fate of food-derived bioactive compounds, including their interaction with the gut microbiota. The gut microbiome plays a crucial role in the metabolism and bioactivation of dietary DNMT inhibitors, potentially modulating their systemic availability and biological effects [26]. Microbial metabolism can generate endogenous epigenetic effectors—such as SCFAs like NaB—that modulate DNMTs and influence DNA methylation patterns. Compounds such as BBR can remodel the microbial composition, thereby indirectly influencing DNA methylation landscapes via host–microbiota crosstalk [26]. This microbiome-epigenome interface adds another layer of complexity—and opportunity—for personalized dietary interventions aimed at epigenetic modulation.

An increasingly promising approach to enhance DNMT inhibition is the combination of bioactive compounds (e.g., SFN + GE + NaB, EGCG + SFN, and others) [51,97,28,61,55]. In summary, the evidence supports the development of synergistic dietary interventions as a promising avenue for epigenetic modulation. Future studies should focus on refining dosages, exploring pharmacokinetics, and optimizing combination strategies to fully harness their preventive and therapeutic potential.

### Minimum effective concentrations of bioactive DNMT modulators: dose considerations, dietary feasibility, and translational insights

A key translational goal of this review was to identify the MECs at which food-derived bioactive compounds modulate DNMT activity. As shown in Table 3, several dietary molecules exhibit significant DNMT-inhibitory effects at low micromolar concentrations. The consistent DNMT-inhibitory effects observed for compounds such as SFN, mahanine, and Annurca apple polyphenols at low micromolar concentrations [43,84,51] support their candidacy for future nutraceutical development. Although multicomponent compounds were excluded from MEC analysis, particularly noteworthy is the enhanced efficacy demonstrated by combinatorial treatments (e.g., EGCG + NaB; EGCG + SFN; SFN + GE + NaB; GTPs + SFN) [28,61,97,51,55], which further highlights the synergistic complexity of nutritional epigenetics as a strategic frontier in disease prevention. Although preclinical studies have identified effective concentrations of DNMT inhibitors, a critical step for their clinical translation is determining whether these doses can be realistically achieved through human dietary intake. For example, SFN at 2 to 5 μM—among the most potent DNMT modulators—can be attained in humans through the consumption of approximately 30 to 50 g of raw broccoli sprouts. Similarly, Annurca apple polyphenols showed effective DNMT inhibition at concentrations as low as 2 μM, which can be reached with the intake of 2 apples per day. GE (soy products) is also present in more commonly consumed foods at concentrations that may be sufficient to induce biologically relevant effects. In contrast, some compounds such as mahanine—an ideal candidate because of its low MEC—are not commonly available in Western diets and would likely require standardization into concentrated extracts, whereas other bioactive molecules, including EGCG (green tea), resveratrol (grapes, red wine), and CU (turmeric), require higher concentrations than those typically consumed through diet, suggesting that supplementation or advanced delivery systems may be needed to achieve therapeutic concentrations in human tissues. Interestingly, folate, a key methyl donor found in leafy greens, represents a particular case in which deficiency, rather than abundance, inhibits DNMT activity. These practical considerations reinforce the promise of food-based or supplementation epigenetic strategies, while also highlighting the need for further pharmacokinetic studies to bridge the gap between in vitro efficacy and in vivo applicability.

### Dietary DNMT modulators and epigenetic aging: a promising avenue in geroscience

Although most dietary DNMT inhibitors have been studied in cancer models, there is growing interest in their ability to counteract age-related DNA hypermethylation at specific CpG sites, potentially restoring a more youthful epigenetic profile without inducing harmful global hypomethylation. Compounds such as resveratrol, CU, and BBR have been shown to modulate DNMT activity [100] while acting on multiple aging pathways, highlighting their potential as dietary geroprotectors.

Recent studies underscore the role of diet in modulating biological age indicators and epigenetic clocks [101]—such as those developed by Horvath [102] and Hannum et al. [103]—which reflect age-related DNA methylation patterns more closely associated with morbidity and mortality than chronological age [20,21,104]. Targeted reprogramming of methylation at key loci may slow epigenetic aging and extend healthspan in aged organisms [105], as also suggested by our findings in both healthy individuals and patients post myocardial infarction, in whom an intensive 60-d relaxation program reduced DNAmAge and improved metabolic and vascular parameters [22]. Similarly, daily supplementation with *M. didyma L.* extract in aging workers stabilized the epigenetic age and improved quality of life and physiological markers [23].

Moreover, the gut microbiota has emerged as a modulator of epigenetic aging. Microbial metabolites, such as SCFAs, derived from dietary substrates, can influence DNMT expression and function. However, whether specific diets can reprogram the host–microbiota–epigenome axis to affect DNAmAge remains an open and compelling question.

To move toward clinical application, future research should focus on the following: *1*) characterizing the pharmacokinetics and tissue distribution of dietary DNMT modulators; *2*) conducting longitudinal intervention trials to evaluate their effects on DNAmAge and healthspan; and *3*) integrating microbiome and epigenetic profiling to develop personalized, nutrition-based anti-aging strategies. These steps are crucial for translating nutritional epigenetics from theoretical promise to practical geroscience.

### Strengths and limitations

This review provides a comprehensive and structured synthesis of the current preclinical evidence on dietary bioactive compounds as modulators of DNMTs, with potential applications in cancer prevention, aging, and epigenetic-based therapies. One of the main strengths lies in the systematic approach adopted, in accordance with the PRISMA guidelines [25], which ensured rigorous selection, screening, and classification of 76 relevant studies. The categorization of compounds into bioactive dietary molecules, natural extracts, and multicomponent compounds proved to be an effective framework for interpreting heterogeneous data and identifying distinct mechanistic profiles and translational opportunities.

A further strength is the integration of mechanistic, structural, and dose-response insights across multiple experimental models. The review highlights not only the prevalence of DNMT inhibition—reported in nearly 90% of studies—but also the molecular underpinnings, including direct enzyme binding, transcriptional regulation, proteasome-mediated degradation, and interactions with methyl donors or transcription factors. The comparative analysis of MECs adds practical value, providing initial reference points for nutraceutical development.

However, several limitations must be acknowledged. The most critical is the preclinical nature of the majority of studies, with 82.9% conducted in vitro, primarily using cancer cell lines. Although these models offer valuable mechanistic insights, they do not fully capture the complexity of human physiology, including issues of bioavailability, metabolism, tissue specificity, and interindividual variability. In vivo and ex vivo studies remain underrepresented, and clinical trials specifically assessing DNMT modulation by dietary compounds are currently lacking. Some included studies were also affected by methodological limitations related to incomplete descriptions of experimental protocols. Although these studies constituted a small proportion (6.6%) and were retained because of consistency with the review’s outcome domain, they introduce a degree of uncertainty that must be considered in interpreting the overall findings. Furthermore, although some studies were also affected by an insufficient investigation of mechanisms of action, the compound investigated in most cases was the same as in other studies that provided mechanistic insights. This supports the plausibility of similar effects. Consequently, these studies were considered compatible with our outcome domain and were included in the review. DNMT modulation or inhibition remained the predominant effect observed across studies, confirming the robustness of the findings despite variations in study quality.

Another key challenge is the variability in compound specificity and activity. Although some agents (e.g., EGCG, CU, GE) show selective inhibition of DNMT1, others affect multiple isoforms (e.g., DNMT3A, DNMT3B) depending on cell type and experimental context. This underscores the need for more precise characterization of target engagement and context-dependent activity.

The determination of minimum effective doses also presents challenges. Although feasible for isolated dietary bioactive compounds tested across multiple studies, it was not possible for natural extracts or multicomponent compounds, which were typically evaluated in single experimental settings. Moreover, potential competitive, antagonistic, or nonlinear interactions among compounds in combined compositions complicate the attribution of effects and the definition of dose thresholds.

Nevertheless, the synergistic potential of certain combinations—such as SFN with NaB or EGCG with GE—emerged as a particularly promising avenue for research. These findings support a shift toward multicomponent, multitarget strategies in nutritional epigenetics, although further investigation is required to determine optimal combinations, dosing regimens, and long-term safety.

## Conclusion

This review sheds new light on DNMTs as key epigenetic targets of dietary bioactive compounds, highlighting a promising and still underexplored avenue in the field of nutritional epigenetics. By systematizing diverse experimental findings, we bring attention to a compelling body of evidence that supports the ability of food-derived bioactive molecules to modulate DNMT activity—an essential mechanism in gene regulation and disease prevention. Beyond confirming the biological plausibility of these effects, our analysis lays the groundwork for a new generation of nutraceutical strategies aimed at epigenetic health promotion. To translate this potential into tangible outcomes, future studies should prioritize the following: *1*) pharmacokinetic investigations to better understand tissue distribution and metabolic dynamics; *2*) long-term dietary intervention trials to evaluate sustained impacts on DNA methylation patterns; and *3*) personalized approaches that integrate epigenetic and microbiome profiling to tailor interventions based on individual biological signatures. By embracing these research directions, we can move from molecular insight to real-world application, paving the way for innovative, noninvasive tools in precision prevention, healthy aging, and sustainable health strategies grounded in everyday nutrition.

## Author contributions

The authors’ responsibilities were as follows – LC, MC, SP: designed the research; LC, MC, SP: conducted the research and wrote the original article; LC, MC, SP: were responsible for final content; LC, MC, SP, FV: revised and edited the article; and all authors: read and approved the final manuscript.

## Data availability

Data described in the manuscript will be made available upon request pending.

## Funding

This work was performed within the framework funded by the European Union—Next Generation EU, in the context of the National Recovery and Resilience Plan, Investment PE8—Project Age-It: “Ageing Well in an Ageing Society” [DM 1557 11.10.2022]. This study was also supported by the funding Grants “2024DCTV1SIDPROGETTI-00194,” provided by the University of Padova, Department of Cardiac, Thoracic, Vascular Science and Public Health.

## Conflict of interest

The authors report no conflicts of interest.
