# Supplementary material for: A Systematic Review of Food-Derived DNA Methyltransferase Modulators: Mechanistic Insights and Perspectives for Healthy Aging
Source: Adv Nutr. 2025 Sep 18;16(11):100521. doi: 10.1016/j.advnut.2025.100521 (PMC12554032; doi:10.1016/j.advnut.2025.100521)
Supplement: multimedia component 1 [file mmc1.docx]

**Supplementary Material**

**A Systematic Review of Food-derived DNA Methyltransferase Modulators: Mechanistic Insights and Perspectives for Healthy Aging**

Manuela Campisi^1,†^, Luana Cannella^1,†^, Francesco Visioli^2^ and Sofia Pavanello ^1,3,4^

^1^ Department of Cardiac, Thoracic, and Vascular Sciences and Public Health, University of Padua, Padua, Italy.

^2^ Department of Molecular Medicine, University of Padua, Padua, Italy

^3^ University Hospital of Padua, Occupational Medicine Unit, Padua, Italy.

^4^ Centre of Studies and Activities for Space CISAS "G. Colombo" of University of Padua, Padua, Italy.

^†^ Contributed equally

ORCID:

Manuela Campisi, 0000-0002-7372-4136

Luana Cannella, 0000-0002-3827-3632

Francesco Visioli, 0000-0002-1756-1723

Sofia Pavanello, 0000-0002-5229-9900

*Correspondence: Sofia Pavanello, sofia.pavanello@unipd.it

**Supplementary Results**

**Dietary molecules, extracts and multicomponent compounds that affect DNA methylation by directly or indirectly acting on the DNMT enzyme.**

Epigenetic modifications, particularly DNA methylation, play a crucial role in regulating gene expression, with DNMTs being the primary enzymes responsible for catalyzing this process. The ability of dietary molecules and natural extracts to influence DNMT activity has gained increasing attention due to its potential implications in disease prevention and therapy (1,2). These bioactive compounds exert their effects through direct inhibition of DNMT catalytic activity or indirect modulation via metabolic pathways, ultimately impacting DNA methylation patterns.

The findings from this review reinforce the role of dietary molecules in epigenetic regulation through DNMT modulation. However, despite a substantial body of preclinical evidence, the lack of clinical trials and the limited validation in in vivo models highlight the need for further translational research. Expanding investigations in animal models and clinical studies will be essential to confirm the efficacy, safety, and therapeutic potential of dietary DNMT modulators for disease prevention and health optimization.

This chapter systematically categorizes the natural compounds identified in the literature into three groups:

1. Dietary molecules – individual bioactive compounds with DNMT-modulating effects.
2. Natural extracts – complex plant-based extracts containing multiple bioactive components.
3. Multicomponent compounds – synergistic combinations of dietary molecules, extracts, or both, which enhance DNMT inhibition.

Each category will be analyzed in detail, outlining biological effects, mechanisms of action, and the minimum effective concentrations required to modulate DNMT activity.

**Dietary molecules: biological effects and mechanism of action on DNMTs**

Each of the food molecules is listed below in alphabetical order with a description of its role in modulating the DNMT enzyme, if it has been tested and evaluated in at least two or more studies.

**Berberine (BBR)**

BBR, a natural alkaloid from Chinese herbal medicine and rhizomes, has demonstrated epigenetic effects in various cancer models. In vitro, treatment with 280 μmol/L in HT29 colorectal adenocarcinoma cells for 48 hours led to downregulation of DNMT1 and DNMT3B mRNA levels, suggesting a role in DNMT regulation potentially by microbiota modulation (3). Ex vivo, in colon tissues from neonatal rats, 10 μM BBR coupled with 15 μM evodiamine altered miRNA expression directly binding the DNMT1, DNMT3A and DNMT3B transcript and post-transcriptionally, leading to time-dependent regulation of DNMT1, DNMT3A, and DNMT3B—initial downregulation at 24h, upregulation at 48h, and DNMT1-specific suppression at 72h (4). In combined studies, BBR inhibited expression and protein of DNMT1 in non‐small cells lung cancer -NSCLC (A549 and H1975) growth in vitro in a dose-dependent manner (6.25-100 μM, 24h), and reduced DNMT1 protein expression in vivo in lung cancer tissues from mice treated with 10 mg/kg. This effect on DNMT1 gene expression is mediated through the Specificity Protein 1 (Sp1) and 3-Phosphoinositide-Dependent Protein Kinase-1 (PDPK1) inhibition (5).

**Curcumin (CU)**

CU, a bioactive compound from turmeric (*Curcuma longa* L*.*), exerts epigenetic effects by targeting DNMT enzyme in cancer models. In vitro, in prostate cancer cells (LNCaP), treatment with 5 μM CU did not alter DNMT1 or DNMT3A protein levels, but it may still contribute to DNA demethylation through enzymatic inhibition instead of acting through protein expression (6). In combined studies, CU treatment in acute myeloid leukemia (AML) cell lines (K562, MV4–11, HL-60) at 5-20 μM (24-72h) in vitro, and 100 mg/kg intraperitoneally in mice (5 days per week for 4 weeks), led to a dose-dependent downregulation of DNMT1 expression via both a chemical inhibitor blocking the catalytic thiol group of DNMT1, and transcriptional modulator through down-modulation of Sp1 and Nuclear factor kappaB (NF-kB) component, and p65 which physically interact and bind to the DNMT1 promoter (7).

**Epigallocatechin gallate (EGCG)**

EGCG, a polyphenol from green tea, exerts epigenetic effects through direct and indirect action on DNMT. In vitro, treatment with 25 μM EGCG (up to 72 hours) in human cervical carcinoma (HeLa) cells led to a time-dependent reduction of DNMT activity and DNMT3B expression through direct binding to the DNMT3B substrate binding pocket (8). In HT29 and HCT116 colon cancer cells, 50-150 μM EGCG (48-72 hours) significantly downregulated DNMT1, DNMT3A, and DNMT3B mRNA levels and induced DNMT3A protein degradation in a dose- and time-dependent manner, directly binding the active site of DNMTs and indirectly reducing the association between DNMT3A and the E3 ubiquitin ligase, Ubiquitin like with PHD and ring finger domains 1 (UHRF1), in methylation-sensitive HCT116 cells (9). Similarly, EGCG, at a concentration ranging from 10 to 20 μg/ml for 3-6 days, revealed a concentration-dependent decrease of DNMT activity, with significant downregulation of all DNMT1, DNMT3A, and DNMT3B at both mRNA and protein levels in epidermoid carcinoma cells (A431, SCC13) (10). This result is in line with what Meeran et al. (11) found in MCF7 and MDA-MB-231 breast cancer cells, treated with 40μM EGCG, showing a direct inhibition of DNMTs activity after 6 and 9 days of treatment due to a strong correlation with the active site residues. Further in vitro study demonstrated that treatment with various concentrations of EGCG decreased DNMTs activity after 7 days treatment in LNCaP cells (12). Wong et al. (13) further confirmed these findings, showing that treatment of Jurkat T cells with EGCG (10 μM and 50 μM) significantly reduced DNMT1, DNMT3A, and DNMT3B mRNA expression. The authors reported that EGCG directly binds to the DNMT substrate binding pocket, thereby exerting its inhibitory effect on DNMT activity. Differently, EGCG failed to induce significant effects on DNA methylation in a study conducted by Stresemann et al. (14) on lymphoid (TK6 and Jurkat), myeloid (KG-1) and colorectal (HCT116) human cancer cell lines.

In vivo, EGCG topical application via a hydrophilic cream to SKH-1 hairless mice reduced DNMT1 activity in UVB-irradiated skin by 41% as compared to normal skin, suggesting a role in methylation regulation by affecting the supply of methyl groups for SAM formation and/or modifying the utilization of methyl groups through processes involving shifts in DNMT activity (15).

**Folate**

Folate, a water-soluble B vitamin found in green leafy vegetables, legumes, fruits, eggs, and grains, plays a crucial role in DNMT regulation and DNA methylation. In primary tumor cells (U373), 4-40 μg/mL folate (7 days) upregulated *DNMT3A* and *DNMT3B* expression through Sp1/Sp3-mediated transcriptional activation, increasing global DNA methylation (16). Further study in colon adenocarcinoma (HCT116, Caco-2), mouse fibroblast (NIH/3T3) and chinese hamster ovary (CHO-K1) cells showed that folate deficiency for 12-20 days led to 28% lower DNMT activity in NIH/3T3 and in Caco-2, and to a significant reduction in DNMT1 and DNMT3A protein levels in NIH/3T3, HCT116 and Caco-2 cells compared to folate-sufficient controls (17). Authors attributed DNMT inhibition under folate-deficient conditions to the lack of SAH-mediated regulation (17). Ex vivo, in brain cortical tissue of F344 rats, a folate-deficient diet (18-36 weeks) resulted in a ~50% reduction in DNMT1 protein levels, while DNMT3A levels increased at both 18 and 36 weeks, and DNMT3B levels increased only at 18 weeks compared to age-matched control rats (18).

**Gallic acid (GA)**

Gallic acid (GA), a polyphenol found in gallnuts, tea leaves, grapes, and pomegranates, influences DNMT activity through distinct mechanisms. In vitro, treatment with 10 μM GA for 7 days in non-small cell cancer cell lines (A549, H1299) led to a significant reduction in DNMT1 and DNMT3B protein levels by negatively regulating protein kinase B (Akt) phosphorylation, thereby reducing DNMT1 nuclear import and protein stability (19). In cardiovascular and cerebrovascular endothelial cells (EAhy926, HBEC-5i), pre-treatment with GA (10-100 μM, 4 hours) restored DNMT1 expression levels by inhibiting protein degradation following exposure to DL-homocysteine (DL-Hcy), adenosine (Ado), and tumor necrosis factor (TNF) as opposed to increased expression of DNMT1 protein (20).

**Genistein (GE)**

GE, a phytoestrogen found in soybeans and fava beans, has been studied for its epigenetic regulation through DNMT activity in several cancer models. In vitro, treatment with 50 μM GE for 48 hours in HeLa cells led to a significant downregulation of *DNMT1*, *DNMT3A*, and *DNMT3B* expression, with a 48% reduction in DNMT activity compared to control cells (21). In MCF-7 and MDA-MB-231, treatment with 60-100 μM GE for 24-72 hours resulted in a decrease in DNMT activity and a dose- and time-dependent decrease in DNMT1 expression and protein levels, while DNMT3A and DNMT3B were unaffected (22). Consistent with these findings, treatment of breast precancerous MCF10AT cells and cancer MCF-7 cells with 50 μM and 100 μM GE respectively for 3 days significantly reduced DNMT1, DNMT3A, and DNMT3B expression in a time-dependent manner in MCF-7, while only DNMT1 was downregulated in MCF10AT cells (23). GE directly interact with the catalytic domain of DNMT1, competitively inhibiting the binding of hemimethylated DNA to its catalytic domain (22). Further studies in LNCaP and PC3 cells and renal carcinoma cell lines (A498, ACHN, HEK-293) confirmed that 50 μM GE reduced DNMT1, DNMT3A and DNMT3B protein levels, effectively downregulating DNMT activity (24,25). In esophageal cancer cells (KYSE 510), 20-100 μM GE inhibited DNMT activity in a dose-dependent manner and in a substrate- and methyl donor–dependent way (26).

In vivo, treatment with 2 mg GE in BALB/c nude mice resulted in DNMT3B downregulation, while the isoforms DNMT1 and DNMT3A levels remained unchanged, suggesting GE’s role as a DNMT inhibitor via chromatin modification (27).

**Mahanine**

Mahanine, from *Micromelum minutum*, decreased DNMT activity in a dose-dependent manner in JB6 P+cells when treated with 2.9-8.6 μM for 3 days (28). The same cells treated with a higher dose (10-20 μM) of mahanine for a shorter time (24-48 h) showed DNMT1 and DNMT3B protein degradation via the ubiquitin-proteasome pathway, reducing their nuclear localization and cytoplasmic staining, while DNMT3A remained unaffected (29). No change in their message levels was detected, suggesting that the decrease in DNMT1 and DNMT3B protein levels occurs post-translationally (29).

**Quercetin**

Quercetin, a flavonoid found in onions, grapes, berries, citrus fruits, vegetables, nuts, propolis, tea, and red wine, influences DNMT activity in different cancer models. In vitro, treatment with 25 and 50 μM quercetin for 48 hours in HeLa cells resulted in a dose-dependent DNMTs inhibition (32-49%) and downregulation of *DNMT1*, *DNMT3A*, and *DNMT3B* expression via competitive inhibition of DNMT3A/3B and suppression of the phosphoinositide 3-kinase (PI3K)-AKT and Wingless-related integration site (WNT) pathways that stabilise DNMT1 (30). In gastric carcinoma cells (SNU719), 45 μM quercetin almost eliminated DNMT1 and DNMT3A expression in a Signal transducer and activator of transcription 3 (STAT3)-indipendent and -dependent manner, respectively, compared to isoliquiritigenin from licorice, which did not affect their expression (31).

In a combined study, treatment with 50-75 μM quercetin for 48-72 hours in AML cell lines (HL60, U937) and human AML xenograft models led to a dose- and time-dependent reduction of DNMT1 and DNMT3A expression, at the protein and message levels, mediated by STAT3 inhibition, a transcription factor that also regulates DNMT (32).

**Resveratrol**

Resveratrol, a polyphenol found in grapes, berries, peanuts, and pines, has been studied for its DNMT inhibitory activity in cancer models. In vitro, pre-treatment or co-treatment with 5-20 μM resveratrol reduced DNMT1 protein levels acting in the BRCA-1 promoter in MCF-7 cells treated with 2,3,7,8-tetrachlorodibenzo-p-dioxin (TCDD), counteracting aryl hydrocarbon receptor (AhR)-mediated BRCA-1 silencing (33). These findings highlight resveratrol’s role in DNMT inhibition other than in chromatin remodelling, supporting its potential in epigenetic cancer therapy (33). Further studies in ERα-negative breast cancer cells (MDA-MB-157, HCC1806) reported the potential of resveratrol, alone or in combination with pterostilbene ̶ found in blueberries and grapes ̶ to downregulate DNMT1, DNMT3A, and DNMT3B expression, with a significant decrease in DNMTs activity (34,35).

It is important to emphasise that resveratrol is not bioavailable and, therefore, its activities and effects in humans remain to be fully elucidated (36).

**Sulforaphane (SFN)**

SFN, a natural compound found in cruciferous vegetables such as broccoli, cabbage, garden cress, cauliflower and Brussels sprouts, has been extensively studied for its DNMT inhibitory effects. In vitro, treatment with 5-20 μM SNF (24 h) in human breast cancer cells (MCF-7, MDA-MB-231, SK-BR-3) led to a decrease in DNMT1, DNMT3A, and DNMT3B protein levels, with a specific reduction in DNMT3A mRNA expression in MDA-MB-231 cells (37). Similarly, Meeran et al. (38) found that 5-20 μM SFN (15 days) inhibited DNMT1 and DNMT3A expression by 62-81% in MCF-7 and by 48-78% in MDA-MB-231 cells; in contrast, no evidence was found by Lubecka-Pietruszewska et al. (39). In HeLa cells, 2.5 μM SNF (24-72 h) induced a time-dependent DNMT inhibition (7-23%) and a time-dependent reduction in DNMT3B expression levels by directly binding to the DNMT3B substrate pocket and preventing the entry of the natural ligand into the active site (40). Further studies in prostate cancer (TRAMP C1) and skin fibroblast (JB6 P+) cells showed that 1-5 μM SFN for 5 days significantly reduced DNMT1, DNMT3A, and DNMT3B protein expression in a dose-dependent manner (41,42), since SFN may interfere with the formation of a transcriptional regulator complex, consisting of methyl-CpG binding proteins, DNMT and histone deacetylase (HDAC) (41). In benign prostate hyperplasia (BPH-1) and prostate cancer (LnCap, PC3) cells, 15-30 μM SFN (24-48 h) decreased DNMT1 and DNMT3A mRNA expression in all cells and DNMT1 protein levels only in LnCap cells, which remained unchanged in BPH-1 and PC3 (43). Additionally, in Caco-2 cells, 50 μM SFN led to a 1.8-fold reduction in DNMT1 expression (44). These effects could be due to SFN's potential intervention in the alternative post-transcriptional and post-translational regulation of DNMTs, in addition to the transcriptional regulation of DNMTs (43).

In a combined study, a 26% SFN-rich broccoli sprout diet in Her2/neu breast cancer mouse models resulted in DNMT1 and DNMT3A downregulation at both mRNA and protein levels, supporting its role in epigenetic modulation; while 5 μM of SFN for 72h was shown to inhibit the protein levels of DNMTs in MDA-MB-157 and MDA-MB-231 cells in *vitro* (45). In a complementary in vitro model, Fan et al. (2012) demonstrated that treatment of porcine satellite cells with 10–15 μM of SFN for 48 hours significantly reduced DNMT1 expression, further confirming the epigenetic modulatory potential of SFN in non-cancerous cell systems (46).

**Other dietary molecules**

Several other dietary bioactive compounds, although less studied because each compound is reported in only one study, have been shown to modulate DNMT activity.

Apigenin (API), found in chamomile flowers, reduced the expression of all DNMT proteins, especially DNMT1 and DNMT3B, in JB6 P+ cells when treated with 1.56-6.5 μM API for 5 days (47).

Dioscin (DS), a saponin compound found in *Dioscorea villosa*, *Saponaria officinalis*, *Quillaja saponaria*, and *Sapindus mukorossi*, was tested in MCF-7, MDA-MB-231 cells. Treatment with 1.15-5.76 μM DS (72 h) did not alter DNMT1, DNMT3A, or DNMT3B in MCF-7 cells but increased DNMT3A mRNA expression in MDA-MB-231 cells at 5.76 μM (48).

Emodin, derived from rhubarb, was studied in pancreatic cancer cells (PANC-1). Treatment with 40 μM emodin (72 h) resulted in a reduction of DNMT1 and DNMT3A mRNA and protein levels, while DNMT3B remained unchanged​ (49).

Parthenolide, derived from *Tanacetum parthenium*, also known as feverfew, was tested in in AML cells (AMV4-11 and Kasumi-1) cells at concentrations ranging from 3.5 to 10 μM and inhibited DNMT1 activity in both cells, while DNMT1 protein was completely depleted in MV4-11 cells and decreased by 50% in Kasumi-1 cells, and mRNA levels decreased by 20% in AMV4-11 cells (50). Parthenolide inhibits DNMT1 both through covalent binding of the catalytic domain, and through the interruption of transcriptional factor Sp1 binding to the promoter of DNMT1 (50).

Peperomin E (PepE), extracted from *Peperomia dindygulensis*, showed a dose-dependent inhibition of DNMT activity in A549 cells with an IC50 of 0.205 μM, and a downregulation of DNMT1 mRNA and protein levels at 4.0 and 8.0 μM (51). PepE directly interacts with the active domain of DNMT1, potentially affecting its activity. This result suggests that the transcriptional repression activity may be due to its ability to reduce the expression of NF-κB (p65) and Sp1, and to reduce the binding of transcription factors to the DNMT1 promoter (51).

Phenethyl isothiocyanate (PEITC), a bioactive compound from cruciferous vegetables such as broccoli and watercress, was investigated in LNCaP cells. Treatment with 1-10 μM PEITC for 1-5 days led to decreased DNMT1, DNMT3A, and DNMT3B mRNA expression, with reduced all DNMTs protein levels at 2.5-5 μM (52).

Trichosanthin (TCS), extracted from the root tubers of *Trichosanthes kirilowi*, was tested in human cervical carcinoma cells (HeLa, CaSki). Treatment with 20-80 μg/ml TCS (48 h) resulted in a dose-dependent decrease of DNMT1 mRNA expression and protein levels, as well as inhibition of enzyme activity (31.3-56.7%)​. The downregulation of DNMT expression and activity is likely due to the nature of TCS as a ribosome-inactivating protein (53).

β-elemene, derived from *Rhizoma zedoariae*, was found to suppress DNMT1 protein expression and cell growth in NSCLC cells (A549, PC9) when tested at 5-60 μg/ml (24 h). This effect was mediated through extracellular signal regulated kinase (ERK1/2)- and AMP-activated protein kinase α (AMPKα)-dependent inhibition of the transcription factor Sp1, followed by reduction in DNMT1 protein expression (54).

Vitamin E showed dose- and context-dependent effects: in vitro, 10 μM increased DNMT1 expression in Caco-2 cells, especially under hyperglycemia (55); in vivo, DNMT1 increased in colon but decreased in liver of mice, depending on diet (56).

**Natural extracts: biological effects and mechanism of action on DNMT**

Natural extracts have demonstrated the ability to modulate DNMT activity, supporting their potential role in epigenetic regulation and cancer prevention.

Annurca polyphenol extract, derived from Annurca apple, was tested in colorectal cancer cells (RKO, SW48, SW480) at 2 μM for 48 hours. While mRNA levels of DNMT1 and DNMT3B remained unchanged, their protein expression was significantly reduced after treatment, suggesting that the extract induces DNMT inhibition at the post-translational level (57).

Black seed oil (or Nigella sativa oil) and its active compound TQ were studied in Jurkat, MCF7, and HeLa cells. Treatment with 0.6-1.25% black seed oil or TQ (10 mM stock, 24 h) resulted in a dose-dependent decrease in DNMT1 mRNA expression, probably due to its direct inhibition of the DNMT1's catalytic pocket, and indirectly through its interaction with the SET and RING-associated (SRA) domain of UHRF1 during DNA replication (58).

BSp, rich in SFN, were investigated in MDA-MB-157 and MDA-MB-231 cells in vitro and in a wild-type (WT) Her2/neu breast cancer mouse model in vivo. In vitro, treatment with 5 μM SFN for 72 h, significantly reduced DNMTs protein levels. Similarly, in vivo, a 26% BSp-enriched diet induced a significant decrease in DNMT1 and DNMT3A expression and their protein levels, but not DNMTs activity (45).

Grape seed proanthocyanidins (GSPs), rich in polyphenols, were tested in A431 and SCC13 cells at 5-20 μg/ml for 3-5 days. GSPs significantly reduced DNMT1, DNMT3A, and DNMT3B mRNA and protein levels, as well as DNMT activity in a dose-dependent manner, with greater inhibition observed after 5 days, suggesting a role in global DNA hypomethylation​ (59).

GTPs, including EGCG, were tested in LNCaP cells at 1-20 μg/ml for 3-14 days. GTPs and EGCG inhibited DNMT1 activity in a dose-dependent manner through the ability of ECGC of block cytosine nucleotide entry into the active site of the DNMT catalytic pocket. GTPs also downregulated DNMT1 mRNA levels and depleted nuclear DNMT1 protein in a dose-dependent manner, with a complete inhibition after 14 days, while DNMT3A and DNMT3B protein levels remained unchanged​ (12).

Consistent with in vitro results, an in vivo study by Henning et al. (60) demonstrated that GTPs significantly inhibited DNMT1 expression in LAPC4 human prostate cancer xenografts. After 13 weeks of oral green tea administration, DNMT1 mRNA and protein levels were reduced by 55% and 40%, respectively, confirming the epigenetic potential of GTPs via both transcriptional and post-translational mechanisms in cancer models. More generally, tea polyphenols (catechin, epicatechin, EGCG) and bioflavonoids (quercetin, fisetin, myricetin) were investigated in prokaryotic bacterial SssI DNMT and human DNMT1 assays, showing that IC50 values of catechin and epicatechin for inhibition of DNMT1 were 4.6 and 8.4 μM, respectively, with the EGCG being the most potent inhibitor (IC50, 0.47 μM), while the IC50 values of quercetin, fisetin, and myricetin for inhibition of the human DNMT1 were 1.6, 3.5, and 1.2 μM, respectively (61). The inhibitory effects of catechin and epicatechin on DNMTs occur through two distinct mechanisms: direct inhibition and indirect inhibition, which results from increased SAH formation during COMT-mediated methylation of these dietary polyphenols. Notably, EGCG exhibits a strong inhibitory effect on human DNMT1, which is further enhanced by Mg²⁺, confirming EGCG as the most potent DNMT’s inhibitor. In contrast, quercetin and fisetin display weak direct inhibition in the absence of COMT, whereas myricetin exerts a much stronger direct inhibitory effect (61).

The methanolic extract of *Paederia foetida* leaves, rich in alkaloids, flavonoids, terpenoids, steroids, cardiac glycosides, and the presence of lupeol and β-sitosterol, was tested in prostate cancer cells (PC-3, DU-145) for 24-72 hours, and resulted in a dose-dependent reduction of DNMT activity and DNMT protein levels (62).

Hop extract and its bioactive compound 6-prenylnaringenin (6-PN), tested in MCF-7 breast cancer cells, were shown to reduce DNMT1-mediated methylation of the CYP1A1 gene after 12 hours of treatment with 5 μg/mL hops extract or 1 μM 6-PN (63). This effect was attributed to activation of AhR, which disrupted the DNMT1-ERα repressive complex, while no changes were observed using anti-DNMT3B antibodies, indicating selective DNMT1 modulation (63).

RAS a traditional Asian medicinal herb, and its active component Z-ligustilide (lig), were tested in TRAMP C1 cells at 8.5 μg/mL (RAS) or 50 μM (Lig) for 3 days. Neither extract altered DNMT1, DNMT3A, or DNMT3B mRNA or protein levels (64).

Wild yam root extract (WYRE) was tested in MCF-7 and MDA-MB-231 cells at 10-30 μg/ml for 72 hours. In MDA-MB-231 cells, WYRE significantly increased DNMT1, DNMT3A, and DNMT3B mRNA expression, while no changes were observed in MCF-7 cells, suggesting a selective epigenetic response in hormone-independent breast cancer​ (48).

**Multicomponent compounds: biological effects and mechanism of action on DNMT**

Some combinations of natural compounds, defined as “multicomponent compounds” ̶ including multiple dietary molecules, extract and dietary molecule, and multiple extracts ̶ , have been studied for their potential synergistic effect on DNMT enzyme and epigenetic modulation in vitro and in vivo models.

1. ***multiple dietary molecules***

Caffeic acid and chlorogenic acid, found in coffee, were observed to act as non-competitive DNMT inhibitors at 5 or 20 µM, increasing SAH levels via COMT-mediated O-methylation, and leading to strong DNMT1 inhibition in a concentration-dependent manner (65).

EGCG, combined with procyanidin B2, inhibited DNMT1 and DNMT3A activity at IC50 values of 9.36 μM and 6.88 μM in MDA-MB-231 cells (66). Another in vitro study demonstrated that EGCG, when combined with NaB ̶ a short-chain fatty acids (SCFAs) produced via gut microbial fermentation ̶ in colorectal cancer cells (RKO, HCT116, HT29), led to a greater reduction in DNMT3A and DNMT3B protein levels, and in DNMT1 expression and protein levels, than when administered alone, inhibiting the catalytic site along with proteasomal degradation (67). Similarly, a combination of EGCG (20 μM) and SFN (10 μM) in MCF-7, MDA-MB-231 and MDA-MB-157 cells, showed a significant reduction in DNMT1 mRNA and protein translation and a decrease in DNMTs enzymatic activity, further enhancing the synergistic effects of polyphenol-rich diets on DNA methylation (68). Supporting these findings, Chen et al. (69) reported that the combination of EGCG (20 μM) and SFN (10 μM) in ovarian cancer SKOV3 cells led to a marked decrease in DNMT1 protein levels, confirming a cooperative epigenetic mechanism through which these compounds may exert anti-cancer effects.

Peyssonenynes A and B, extracted from the Fijian red marine alga *Peyssonnelia caulifera*, were tested in DNMT enzyme assays, and showed a strong DNMT1 inhibition at 16 and 9 μM, respectively (70). Likewise, Psammaplin A, bisaprasin, and psammaplin G, isolated from the marine sponge *Pseudoceratina purpurea*, were tested in solid tumor (Colon38, ColonH116, Lung H125) and leukemia cells (L1210, CEM), indicating that these compounds act as DNMT inhibitors at each IC50 doses (71). Both these studies highlight the potential of marine-derived metabolites​ as epigenetic modulators (70,71).

Resveratrol in combination with pterostilbene ̶ found in blueberries and grapes ̶ has shown to downregulate DNMT1, DNMT3A, and DNMT3B expression in ERα-negative breast cancer cells (MDA-MB-157, HCC1806), with a significant decrease in DNMTs activity (34,35).

GE and daidzein, two soy-derived isoflavones, and biochanin A, the major isoflavones in red clover (*Trifolium pratense*), were tested in KYSE510 cells at different concentrations for 6 days, revealing a dose-dependent inhibition of DNMT activity at higher GE concentration (20-50-100 μmol/L) in a substrate- and methyl donor–dependent manner, while biochanin A and daidzein were less effective (26).

SFN and GE ̶ derived from cruciferous vegetables and soy ̶ and NaB ̶ produced via gut microbial fermentation ̶ were tested in MDA-MB-231 and MCF-7 cells. Treatment with single, double, and triple combinations (5 μM SFN, 15 μM GE, 2.5 mM NaB) significantly downregulated DNMT3A and DNMT3B mRNA and protein levels, with the strongest effect observed in the SFN-GE-NaB combination (72). DNMT activity was also significantly reduced in both breast cancer cells​ by double and triple combinations of these dietary compounds (72).

A combination of EGCG, GE, CU, resveratrol, withaferin A, and guggulsterone, derived from green tea, soy, turmeric, grapes, and medicinal herbs, was tested in MCF7 and MDA-MB-231 cells. Treatment with these compounds at IC50 concentrations for 96 hours significantly downregulated DNMT1, DNMT3A, and DNMT3B expression, with DNMT1 protein levels decreasing 2- to 3-fold​ (73).

A multicomponent mixture containing baicalein, myricetin, protocatechuic acid, phloretin, sinapic acid, syringic acid, resveratrol, rosmarinic acid, ellagic acid, betanin, cyanidin, and galangin, derived from various plants, was tested in MCF7 cells. Authors showed that treatment with 20-40 μM for 3 days resulted in up to 88% inhibition of DNMT activity, with ellagic acid and rosmarinic acid being the most potent inhibitors, largely due to the increased formation of SAH resulting from the COMT-mediated O-methylation (74). Rosmarinic acid alone (20 μM and 40 μM) also showed a 20-30% reduction in DNMT1 protein levels (74).

EGCG, GE, myricetin, quercetin, hesperetin, naringenin, API, luteolin, garcinol, CU, and hydroxycinnamic acid, all dietary polyphenols, tested in KYSE 150, LNCaP and PC3 cells inhibited DNMT activity at 20-50 μM, with EGCG being the most potent, followed by hydroxycinnamic acid, garcinol, and luteolin (≥50% inhibition at 50 μM), and GE being the weaker inhibitor (75). These findings were explained by the direct interaction between EGCG and DNMT with EGCG binding to the enzyme’s active pocket, while the other catechol polyphenols may indirectly inhibit the DNMT enzyme by generating SAH upon their methylation by SAM (75).

Additionally, in an in vivo model, Blagojević et al. (76) evaluated the effects of a multi-nutrient supplement (Espermaplus), containing liposoluble and water-soluble vitamins, omega-3 fatty acids, essential amino acids, and trace elements, administered at 75 g/day for 12 weeks to adult boars. Although this complex combination mirrors a “multicomponent” approach, no significant changes were observed in DNMT3A and DNMT3B expression, suggesting that not all complex formulations exert an epigenetic impact on DNMT regulation (76).

1. ***extract and dietary molecule***

GTPs and SFN, alone and in combination, were used to treat breast cancer cells (MCF-7, MDA-MB-231, MDA-MB-453) for 3 days. While GTPs (20 µM) or SFN (10 µM) inhibited DNMTs activity and expression but only decreased DNMT3A or DNMT1 protein, their combination showed a more pronounced DNMTs inhibitory effect and decreased all DNMT protein levels (77).

1. ***Multiple extracts***

A dietary combination of GTPs and BSp was tested in mouse orthotopic xenografts. Mice fed 0.3% GTPs and 13% BSp for 2 weeks before tumor injection and throughout the study exhibited significant DNMT1 downregulation, reinforcing the synergistic DNMT-inhibitory effects of polyphenol-rich diets (68).

**Determination of the minimum effective dose of dietary DNMT inhibitors**

A key aspect of this study was to establish the minimum effective dose of dietary molecules required to induce DNMT inhibition and epigenetic modifications, providing insight into their potential clinical relevance. A systematic dose-response analysis across multiple studies identified a range of concentrations at which dietary compounds exerted significant DNMT inhibition and altered DNA methylation patterns, particularly for the most investigated molecules in two or more studies (see Table 3).

The lowest effective concentrations varied depending on the compound, experimental model, and mechanism of action. Among the dietary molecules, EGCG (20-25 μM) (8), GE (20-50 μM) (26), quercetin (25-50 μM) (30) and CU (5-20 μM) (7) demonstrated dose-dependent DNMT inhibition at relatively low concentrations. Resveratrol also requires similar concentration (5-20 μM) (33) to exert measurable epigenetic effects on its own, but lower concentrations (5 μM) are needed when studied in combination treatments, in particular with pterostilbene (34,35). SFN (1-5 μM) (41,42) and folic acid deficiency (17), which influence methyl donor metabolism, exhibited broader DNMT modulation, reinforcing their role in methylation homeostasis. BBR (6.25-100 μM) (5), GA (10 μM) (19) and mahanine (2.9-8.6 μM) (28) also emerged as potent DNMT modulators​.

Among the natural extracts, Annurca polyphenol extract (2 μM) (57), black seed oil (0.6-1.25%) (58), GTPs (1-20 μg/ml) (12) and RAS (8.5 μg/ml) (64) demonstrated DNMT inhibition primarily via transcriptional and post-translational mechanisms,​ with the lower doses also taking into account the different unit of measurement used in different studies. However, we cannot define them as the minimum effective dose as each of the natural extracts identified was only studied once.

Several synergistic interactions were further detected by analyzing multiple dietary molecules. Since again each combination was tested in a single study, it is not possible to determine a proper minimum effective dose, but we observed that bioactive molecules such as EGCG, SFN, and GE when used in combination (67,68,72,77), showed a more pronounced inhibitory effect on all isoforms of the DNMT enzyme.

**Supplementary Tables**

**Supplementary Table 1.** Search strategy: keywords used in PubMed database and number of articles published up to May 23, 2025.

| **First term** | **Second term** | **Number of articles** |
| --- | --- | --- |
| Food molecules | DNA methyltransferase | 41 |
|  | DNMT | 14 |
|  | DNA methyltransferase inhibitors | 16 |
|  | DNMT inhibitors | 8 |
| Bioactive dietary compounds | DNA methyltransferase | 13 |
|  | DNMT | 5 |
|  | DNA methyltransferase inhibitors | 7 |
|  | DNMT inhibitors | 3 |

**Supplementary Table 2.** Risk of bias due to unclear methodological descriptions or lacked of detailed investigation of mechanism of action, with number of articles, % and references.

| **Risk of Bias** | **Number of studies (N) and %** | **References** |
| --- | --- | --- |
| Unclear methodological descriptions | N=5, 6.6% | (14,61,65,71,75) |
| Lacked of detailed investigation of mechanism of action | N=36, 47.4% | (6,10,14,18,21,23–25,28,34,35,37–39,42,44–49,52,55–57,59,60,62,64,68–72,76) |

**References**

1. Park LK, Friso S, Choi S-W. Nutritional influences on epigenetics and age-related disease. Proc Nutr Soc 2012;71:75–83.

2. Choi S-W, Friso S. Epigenetics: A New Bridge between Nutrition and Health. Advances in Nutrition 2010;1:8–16.

3. Wang X, Peng A, Huang C. Suppression of colon cancer growth by berberine mediated by the intestinal microbiota and the suppression of DNA methyltransferases (DNMTs). Mol Cell Biochem 2024;479:2131–41.

4. Huang C, Liu H, Gong X-L, Wu L-Y, Wen B. Effect of evodiamine and berberine on the interaction between DNMTs and target microRNAs during malignant transformation of the colon by TGF-β1. Oncology Reports Spandidos Publications; 2017;37:1637–45.

5. Zheng F, Wu J, Tang Q, Xiao Q, Wu W, Hann SS. The enhancement of combination of berberine and metformin in inhibition of DNMT1 gene expression through interplay of SP1 and PDPK1. J Cell Mol Med 2018;22:600–12.

6. Shu L, Khor TO, Lee J-H, Boyanapalli SSS, Huang Y, Wu T-Y, Saw CL-L, Cheung K-L, Kong A-NT. Epigenetic CpG Demethylation of the Promoter and Reactivation of the Expression of Neurog1 by Curcumin in Prostate LNCaP Cells. AAPS J 2011;13:606–14.

7. Yu J, Peng Y, Wu L-C, Xie Z, Deng Y, Hughes T, He S, Mo X, Chiu M, Wang Q-E, et al. Curcumin Down-Regulates DNA Methyltransferase 1 and Plays an Anti-Leukemic Role in Acute Myeloid Leukemia. PLOS ONE Public Library of Science; 2013;8:e55934.

8. Khan MA, Hussain A, Sundaram MK, Alalami U, Gunasekera D, Ramesh L, Hamza A, Quraishi U. (-)-Epigallocatechin-3-gallate reverses the expression of various tumor-suppressor genes by inhibiting DNA methyltransferases and histone deacetylases in human cervical cancer cells. Oncology Reports Spandidos Publications; 2015;33:1976–84.

9. Moseley VR, Morris J, Knackstedt RW, Wargovich MJ. Green Tea Polyphenol Epigallocatechin 3-Gallate, Contributes to the Degradation of DNMT3A and HDAC3 in HCT 116 Human Colon Cancer Cells. Anticancer Research International Institute of Anticancer Research; 2013;33:5325–33.

10. Nandakumar V, Vaid M, Katiyar SK. (−)-Epigallocatechin-3-gallate reactivates silenced tumor suppressor genes, Cip1/p21 and p 16 INK4a , by reducing DNA methylation and increasing histones acetylation in human skin cancer cells. Carcinogenesis 2011;32:537–44.

11. Meeran SM, Patel SN, Chan T-H, Tollefsbol TO. A Novel Prodrug of Epigallocatechin-3-gallate: Differential Epigenetic hTERT Repression in Human Breast Cancer Cells. Cancer Prevention Research 2011;4:1243–54.

12. Pandey M, Shukla S, Gupta S. Promoter demethylation and chromatin remodeling by green tea polyphenols leads to re-expression of GSTP1 in human prostate cancer cells. International Journal of Cancer 2010;126:2520–33.

13. Wong CP, Nguyen LP, Noh SK, Bray TM, Bruno RS, Ho E. Induction of regulatory T cells by green tea polyphenol EGCG. Immunol Lett 2011;139:7–13.

14. Stresemann C, Brueckner B, Musch T, Stopper H, Lyko F. Functional Diversity of DNA Methyltransferase Inhibitors in Human Cancer Cell Lines. Cancer Research 2006;66:2794–800.

15. Mittal A, Piyathilake C, Hara Y, Katiyar SK. Exceptionally High Protection of Photocarcinogenesis by Topical Application of (—)-Epigallocatechin-3-Gallate in Hydrophilic Cream in SKH-1 Hairless Mouse Model: Relationship to Inhibition of UVB-Induced Global DNA Hypomethylation. Neoplasia 2003;5:555–65.

16. Hervouet E, Debien E, Campion L, Charbord J, Menanteau J, Vallette FM, Cartron P-F. Folate supplementation limits the aggressiveness of glioma via the remethylation of DNA repeats element and genes governing apoptosis and proliferation. Clin Cancer Res 2009;15:3519–29.

17. Stempak JM, Sohn K-J, Chiang E-P, Shane B, Kim Y-I. Cell and stage of transformation-specific effects of folate deficiency on methionine cycle intermediates and DNA methylation in an in vitro model. Carcinogenesis 2005;26:981–90.

18. Pogribny IP, Karpf AR, James SR, Melnyk S, Han T, Tryndyak VP. Epigenetic alterations in the brains of Fisher 344 rats induced by long-term administration of folate/methyl-deficient diet. Brain Research 2008;1237:25–34.

19. Weng Y-P, Hung P-F, Ku W-Y, Chang C-Y, Wu B-H, Wu M-H, Yao J-Y, Yang J-R, Lee C-H. The inhibitory activity of gallic acid against DNA methylation: application of gallic acid on epigenetic therapy of human cancers. Oncotarget Impact Journals; 2017;9:361–74.

20. Kam A, Li KM, Razmovski-Naumovski V, Nammi S, Chan K, Li GQ. Gallic acid protects against endothelial injury by restoring the depletion of DNA methyltransferase 1 and inhibiting proteasome activities. International Journal of Cardiology 2014;171:231–42.

21. Sundaram MK, Unni S, Somvanshi P, Bhardwaj T, Mandal RK, Hussain A, Haque S. Genistein Modulates Signaling Pathways and Targets Several Epigenetic Markers in HeLa Cells. Genes (Basel) 2019;10:955.

22. Xie Q, Bai Q, Zou L-Y, Zhang Q-Y, Zhou Y, Chang H, Yi L, Zhu J-D, Mi M-T. Genistein inhibits DNA methylation and increases expression of tumor suppressor genes in human breast cancer cells. Genes, Chromosomes and Cancer 2014;53:422–31.

23. Li Y, Liu L, Andrews LG, Tollefsbol TO. Genistein depletes telomerase activity through cross-talk between genetic and epigenetic mechanisms. International Journal of Cancer 2009;125:286–96.

24. Majid S, Dar AA, Shahryari V, Hirata H, Ahmad A, Saini S, Tanaka Y, Dahiya AV, Dahiya R. Genistein reverses hypermethylation and induces active histone modifications in tumor suppressor gene B-Cell translocation gene 3 in prostate cancer. Cancer 2010;116:66–76.

25. Majid S, Dar AA, Ahmad AE, Hirata H, Kawakami K, Shahryari V, Saini S, Tanaka Y, Dahiya AV, Khatri G, et al. BTG3 tumor suppressor gene promoter demethylation, histone modification and cell cycle arrest by genistein in renal cancer. Carcinogenesis 2009;30:662–70.

26. Fang MZ, Chen D, Sun Y, Jin Z, Christman JK, Yang CS. Reversal of Hypermethylation and Reactivation of p16INK4a, RARβ, and MGMT Genes by Genistein and Other Isoflavones from Soy. Clinical Cancer Research 2005;11:7033–41.

27. Li H, Xu W, Huang Y, Huang X, Xu L, Lv Z. Genistein demethylates the promoter of CHD5 and inhibits neuroblastoma growth in vivo. International Journal of Molecular Medicine Spandidos Publications; 2012;30:1081–6.

28. Jagadeesh S, Sinha S, Pal BC, Bhattacharya S, Banerjee PP. Mahanine reverses an epigenetically silenced tumor suppressor gene RASSF1A in human prostate cancer cells. Biochemical and Biophysical Research Communications 2007;362:212–7.

29. Agarwal S, Amin KS, Jagadeesh S, Baishay G, Rao PG, Barua NC, Bhattacharya S, Banerjee PP. Mahanine restores RASSF1A expression by down-regulating DNMT1 and DNMT3B in prostate cancer cells. Molecular Cancer 2013;12:99.

30. Sundaram M, Hussain A, Haque S, Raina R, Afroze N. Quercetin modifies 5′CpG promoter methylation and reactivates various tumor suppressor genes by modulating epigenetic marks in human cervical cancer cells. Journal of Cellular Biochemistry 2019;120:18357–69.

31. Lee M, Son M, Ryu E, Shin YS, Kim JG, Kang BW, Cho H, Kang H. Quercetin-induced apoptosis prevents EBV infection. Oncotarget Impact Journals; 2015;6:12603–24.

32. Alvarez MC, Maso V, Torello CO, Ferro KP, Saad STO. The polyphenol quercetin induces cell death in leukemia by targeting epigenetic regulators of pro-apoptotic genes. Clinical Epigenetics 2018;10:139.

33. Papoutsis AJ, Lamore SD, Wondrak GT, Selmin OI, Romagnolo DF. Resveratrol Prevents Epigenetic Silencing of BRCA-1 by the Aromatic Hydrocarbon Receptor in Human Breast Cancer Cells1,2. The Journal of Nutrition 2010;140:1607–14.

34. Kala R, Shah HN, Martin SL, Tollefsbol TO. Epigenetic-based combinatorial resveratrol and pterostilbene alters DNA damage response by affecting SIRT1 and DNMT enzyme expression, including SIRT1-dependent γ-H2AX and telomerase regulation in triple-negative breast cancer. BMC Cancer 2015;15:672.

35. Kala R, Tollefsbol TO. A Novel Combinatorial Epigenetic Therapy Using Resveratrol and Pterostilbene for Restoring Estrogen Receptor-α (ERα) Expression in ERα-Negative Breast Cancer Cells. PLOS ONE Public Library of Science; 2016;11:e0155057.

36. Tang PC-T, Ng Y-F, Ho S, Gyda M, Chan S-W. Resveratrol and cardiovascular health--promising therapeutic or hopeless illusion? Pharmacol Res 2014;90:88–115.

37. Lewinska A, Adamczyk-Grochala J, Deregowska A, Wnuk M. Sulforaphane-Induced Cell Cycle Arrest and Senescence are accompanied by DNA Hypomethylation and Changes in microRNA Profile in Breast Cancer Cells. Theranostics Ivyspring International Publisher; 2017;7:3461–77.

38. Meeran SM, Patel SN, Tollefsbol TO. Sulforaphane Causes Epigenetic Repression of hTERT Expression in Human Breast Cancer Cell Lines. PLOS ONE Public Library of Science; 2010;5:e11457.

39. Lubecka-Pietruszewska K, Kaufman-Szymczyk A, Stefanska B, Cebula-Obrzut B, Smolewski P, Fabianowska-Majewska K. Sulforaphane Alone and in Combination with Clofarabine Epigenetically Regulates the Expression of DNA Methylation-Silenced Tumour Suppressor Genes in Human Breast Cancer Cells. Journal of Nutrigenetics and Nutrigenomics 2015;8:91–101.

40. Khan M, Kedhari Sundaram M, Hamza A, Quraishi U, Gunasekera D, Ramesh L, Goala P, Al Alami U, Ansari MZ, Rizvi TA, et al. Sulforaphane Reverses the Expression of Various Tumor Suppressor Genes by Targeting DNMT3B and HDAC1 in Human Cervical Cancer Cells. Evidence-Based Complementary and Alternative Medicine 2015;2015:412149.

41. Zhang C, Su Z-Y, Khor TO, Shu L, Kong A-NT. Sulforaphane enhances Nrf2 expression in prostate cancer TRAMP C1 cells through epigenetic regulation. Biochemical Pharmacology 2013;85:1398–404.

42. Su Z-Y, Zhang C, Lee JH, Shu L, Wu T-Y, Khor TO, Conney AH, Lu Y-P, Kong A-NT. Requirement and Epigenetics Reprogramming of Nrf2 in Suppression of Tumor Promoter TPA-Induced Mouse Skin Cell Transformation by Sulforaphane. Cancer Prevention Research 2014;7:319–29.

43. Hsu A, Wong CP, Yu Z, Williams DE, Dashwood RH, Ho E. Promoter de-methylation of cyclin D2 by sulforaphane in prostate cancer cells. Clin Epigenetics 2011;3:3.

44. Traka M, Gasper AV, Smith JA, Hawkey CJ, Bao Y, Mithen RF. Transcriptome Analysis of Human Colon Caco-2 Cells Exposed to Sulforaphane123. The Journal of Nutrition 2005;135:1865–72.

45. Li S, Chen M, Wu H, Li Y, Tollefsbol TO. Maternal epigenetic regulation contributes to prevention of estrogen receptor-negative mammary cancer with broccoli sprout consumption. Cancer Prev Res (Phila) 2020;13:449–62.

46. Fan H, Zhang R, Tesfaye D, Tholen E, Looft C, Hölker M, Schellander K, Cinar MU. Sulforaphane causes a major epigenetic repression of myostatin in porcine satellite cells. Epigenetics 2012;7:1379–90.

47. Paredes-Gonzalez X, Fuentes F, Su Z-Y, Kong A-NT. Apigenin Reactivates Nrf2 Anti-oxidative Stress Signaling in Mouse Skin Epidermal JB6 P + Cells Through Epigenetics Modifications. AAPS J 2014;16:727–35.

48. Aumsuwan P, Khan SI, Khan IA, Ali Z, Avula B, Walker LA, Shariat-Madar Z, Helferich WG, Katzenellenbogen BS, Dasmahapatra AK. The anticancer potential of steroidal saponin, dioscin, isolated from wild yam (*Dioscorea villosa*) root extract in invasive human breast cancer cell line MDA-MB-231 in vitro. Archives of Biochemistry and Biophysics 2016;591:98–110.

49. Pan F-P, Zhou H-K, Bu H-Q, Chen Z-Q, Zhang H, Xu L-P, Tang J, Yu Q-J, Chu Y-Q, Pan J, et al. Emodin enhances the demethylation by 5-Aza-CdR of pancreatic cancer cell tumor-suppressor genes P16, RASSF1A and ppENK. Oncology Reports Spandidos Publications; 2016;35:1941–9.

50. Liu Z, Liu S, Xie Z, Pavlovicz RE, Wu J, Chen P, Aimiuwu J, Pang J, Bhasin D, Neviani P, et al. Modulation of DNA methylation by a sesquiterpene lactone parthenolide. J Pharmacol Exp Ther 2009;329:505–14.

51. Wang X, Cheng Y, Wang K, Liu R, Yang X, Wen H, Chai C, Liang J, Wu H. Peperomin E reactivates silenced tumor suppressor genes in lung cancer cells by inhibition of DNA methyltransferase. Cancer Science 2016;107:1506–19.

52. Boyanapalli SSS, Li W, Fuentes F, Guo Y, Ramirez CN, Gonzalez X-P, Pung D, Kong A-NT. Epigenetic reactivation of RASSF1A by phenethyl isothiocyanate (PEITC) and promotion of apoptosis in LNCaP cells. Pharmacological Research 2016;114:175–84.

53. Huang Y, Song H, Hu H, Cui L, You C, Huang L. Trichosanthin inhibits DNA methyltransferase and restores methylation-silenced gene expression in human cervical cancer cells. Mol Med Rep 2012;6:872–8.

54. Zhao S, Wu J, Zheng F, Tang Q, Yang L, Li L, Wu W, Hann SS. β-elemene inhibited expression of DNA methyltransferase 1 through activation of ERK1/2 and AMPKα signalling pathways in human lung cancer cells: the role of Sp1. J Cell Mol Med 2015;19:630–41.

55. Zappe K, Pointner A, Switzeny OJ, Magnet U, Tomeva E, Heller J, Mare G, Wagner K-H, Knasmueller S, Haslberger AG. Counteraction of Oxidative Stress by Vitamin E Affects Epigenetic Regulation by Increasing Global Methylation and Gene Expression of MLH1 and DNMT1 Dose Dependently in Caco-2 Cells. Oxid Med Cell Longev 2018;2018:3734250.

56. Remely M, Ferk F, Sterneder S, Setayesh T, Kepcija T, Roth S, Noorizadeh R, Greunz M, Rebhan I, Wagner K-H, et al. Vitamin E Modifies High-Fat Diet-Induced Increase of DNA Strand Breaks, and Changes in Expression and DNA Methylation of Dnmt1 and MLH1 in C57BL/6J Male Mice. Nutrients Multidisciplinary Digital Publishing Institute; 2017;9:607.

57. Fini L, Selgrad M, Fogliano V, Graziani G, Romano M, Hotchkiss E, Daoud YA, De Vol EB, Boland CR, Ricciardiello L. Annurca Apple Polyphenols Have Potent Demethylating Activity and Can Reactivate Silenced Tumor Suppressor Genes in Colorectal Cancer Cells1,2. The Journal of Nutrition 2007;137:2622–8.

58. Alsanosi S, Sheikh RA, Sonbul S, Altayb HN, Batubara AS, Hosawi S, Al-Sakkaf K, Abdullah O, Omran Z, Alhosin M. The Potential Role of Nigella sativa Seed Oil as Epigenetic Therapy of Cancer. Molecules Multidisciplinary Digital Publishing Institute; 2022;27:2779.

59. Vaid M, Prasad R, Singh T, Jones V, Katiyar SK. Grape seed proanthocyanidins reactivate silenced tumor suppressor genes in human skin cancer cells by targeting epigenetic regulators. Toxicol Appl Pharmacol 2012;263:122–30.

60. Henning SM, Wang P, Said J, Magyar C, Castor B, Doan N, Tosity C, Moro A, Gao K, Li L, et al. Polyphenols in brewed green tea inhibit prostate tumor xenograft growth by localizing to the tumor and decreasing oxidative stress and angiogenesis. J Nutr Biochem 2012;23:1537–42.

61. Lee WJ, Shim J-Y, Zhu BT. Mechanisms for the inhibition of DNA methyltransferases by tea catechins and bioflavonoids. Mol Pharmacol 2005;68:1018–30.

62. Pradhan N, Parbin S, Kausar C, Kar S, Mawatwal S, Das L, Deb M, Sengupta D, Dhiman R, Patra SK. Paederia foetida induces anticancer activity by modulating chromatin modification enzymes and altering pro-inflammatory cytokine gene expression in human prostate cancer cells. Food Chem Toxicol 2019;130:161–73.

63. Hitzman RT, Dunlap TL, Howell CE, Chen S-N, Vollmer G, Pauli GF, Bolton JL, Dietz BM. 6-Prenylnaringenin from Hops Disrupts ERα-mediated Downregulation of CYP1A1 to Facilitate Estrogen Detoxification. Chem Res Toxicol 2020;33:2793–803.

64. Su Z-Y, Khor TO, Shu L, Lee JH, Saw CL-L, Wu T-Y, Huang Y, Suh N, Yang CS, Conney AH, et al. Epigenetic Reactivation of Nrf2 in Murine Prostate Cancer TRAMP C1 Cells by Natural Phytochemicals Z-Ligustilide and Radix Angelica Sinensis via Promoter CpG Demethylation. Chem Res Toxicol American Chemical Society; 2013;26:477–85.

65. Lee WJ, Zhu BT. Inhibition of DNA methylation by caffeic acid and chlorogenic acid, two common catechol-containing coffee polyphenols. Carcinogenesis 2006;27:269–77.

66. Shilpi A, Parbin S, Sengupta D, Kar S, Deb M, Rath SK, Pradhan N, Rakshit M, Patra SK. Mechanisms of DNA methyltransferase–inhibitor interactions: Procyanidin B2 shows new promise for therapeutic intervention of cancer. Chemico-Biological Interactions 2015;233:122–38.

67. Saldanha SN, Kala R, Tollefsbol TO. Molecular mechanisms for inhibition of colon cancer cells by combined epigenetic-modulating epigallocatechin gallate and sodium butyrate. Exp Cell Res 2014;324:40–53.

68. Li Y, Meeran SM, Tollefsbol TO. Combinatorial bioactive botanicals re-sensitize tamoxifen treatment in ER-negative breast cancer via epigenetic reactivation of ERα expression. Sci Rep 2017;7:9345.

69. Chen H, Landen CN, Li Y, Alvarez RD, Tollefsbol TO. Epigallocatechin Gallate and Sulforaphane Combination Treatment Induce Apoptosis in Paclitaxel-Resistant Ovarian Cancer Cells through hTERT and Bcl-2 Down-regulation. Exp Cell Res 2013;319:697–706.

70. McPhail KL, France D, Cornell-Kennon S, Gerwick WH. Peyssonenynes A and B, Novel Enediyne Oxylipins with DNA Methyl Transferase Inhibitory Activity from the Red Marine Alga Peyssonnelia caulifera. J Nat Prod American Chemical Society; 2004;67:1010–3.

71. Piña IC, Gautschi JT, Wang G-Y-S, Sanders ML, Schmitz FJ, France D, Cornell-Kennon S, Sambucetti LC, Remiszewski SW, Perez LB, et al. Psammaplins from the Sponge Pseudoceratina purpurea: Inhibition of Both Histone Deacetylase and DNA Methyltransferase. J Org Chem American Chemical Society; 2003;68:3866–73.

72. Sharma M, Tollefsbol TO. Combinatorial epigenetic mechanisms of sulforaphane, genistein and sodium butyrate in breast cancer inhibition. Experimental Cell Research 2022;416:113160.

73. Mirza S, Sharma G, Parshad R, Gupta SD, Pandya P, Ralhan R. Expression of DNA Methyltransferases in Breast Cancer Patients and to Analyze the Effect of Natural Compounds on DNA Methyltransferases and Associated Proteins. Journal of Breast Cancer 2013;16:23–31.

74. Paluszczak J, Krajka-Kuźniak V, Baer-Dubowska W. The effect of dietary polyphenols on the epigenetic regulation of gene expression in MCF7 breast cancer cells. Toxicology Letters 2010;192:119–25.

75. Fang M, Chen D, Yang CS. Dietary Polyphenols May Affect DNA Methylation123. The Journal of Nutrition 2007;137:223S-228S.

76. Blagojević J, Stanimirović Z, Glavinić U, Vakanjac S, Radukić Ž, Mirilović M, Maletić M. Impact of Supplemented Nutrition on Semen Quality, Epigenetic-Related Gene Expression, and Oxidative Status in Boars. Animals (Basel) 2024;14:3297.

77. Meeran SM, Patel SN, Li Y, Shukla S, Tollefsbol TO. Bioactive Dietary Supplements Reactivate ER Expression in ER-Negative Breast Cancer Cells by Active Chromatin Modifications. PLOS ONE Public Library of Science; 2012;7:e37748.
